# Early‐Life Manganese Overexposure Activates Microglial Phagocytosis through the YY1/TREM2 Axis Leading to Hippocampal Synaptic Remodeling in Mice

**DOI:** 10.1002/advs.78036

**Published:** 2026-10-04

**Authors:** Keyu Chen, Bin He, Kuan Liu, Zhuo Ma, Bin Xu

**Affiliations:** ^1^ Key Laboratory of Environmental Stress and Chronic Disease Control and Prevention (China Medical University) Ministry of Education Shenyang Liaoning P. R. China; ^2^ Program of Environmental Physical Factors and Health School of Public Health China Medical University Shenyang Liaoning P. R. China; ^3^ Department of General Surgery Shengjing Hospital of China Medical University Shenyang Liaoning P. R. China

**Keywords:** hippocampal development, microglial phagocytosis, neurological disorders, triggering receptor expressed on myeloid cells 2 (TREM2), yin yang 1 (YY1)

## Abstract

The early‐stage hippocampal development depends on precise synaptic remodeling, processes where microglia play indispensable roles. Disruption of this event reshape hippocampal neural circuit architecture and heighten the risk for long‐term memory, learning, and behavioral deficits. Manganese (Mn), a widespread environmental contaminant, is recognized for neurodevelopmental toxicity and reported disrupting hippocampal synaptic integrity. Here, we report that early‐life Mn exposure impaired learning, long‐term memory, and behavioral performance in young adulthood male mice, accompanied by microglia excessive‐phagocytosis and aberrant hippocampal synaptic remodeling. Single‐nucleus transcriptomics, CUT&Tag profiling collectively reveal that Mn exposure elevated the transcription factor Yin Yang 1 (YY1) expression in developing microglia and directly activated transcription of triggering receptor expressed on myeloid cells 2 (TREM2) and phagolysosomal pathway‐related genes, thereby driving excessive microglial phagocytosis. This heightened engulfment capacity led to over‐phagocytosis ultimately resulting in abnormal synaptic remodeling during critical developmental windows. Mechanistically, Mn‐induced YY1 accumulation may result from enhanced HIF1α‐mediated transcription and disrupted SMURF2‐dependent ubiquitin degradation. Together, our findings identified YY1/TREM2 as an axis through which early‐life Mn exposure perturbed microglial homeostasis and induced maladaptive synaptic remodeling. These results provide mechanistic insight into neurodevelopmental consequences of Mn exposure and highlight potential molecular targets for potential early therapeutic intervention of neurological disorders.

## Graphical abstract

Early‐life manganese exposure induces behavioral deficits and aberrant hippocampal synaptic remodeling by enhancing microglial phagocytosis in young adult male mice. Mechanistically, manganese promotes Yin Yang 1 (YY1)/triggering receptor expressed on myeloid cells 2 (TREM2) signaling through enhanced HIF1α‐mediated transcription and suppressed SMURF2‐mediated degradation of YY1. These findings reveal that manganese exposure reshapes microglial function, which contributes to neurobehavioral abnormalities in young adult male mice.

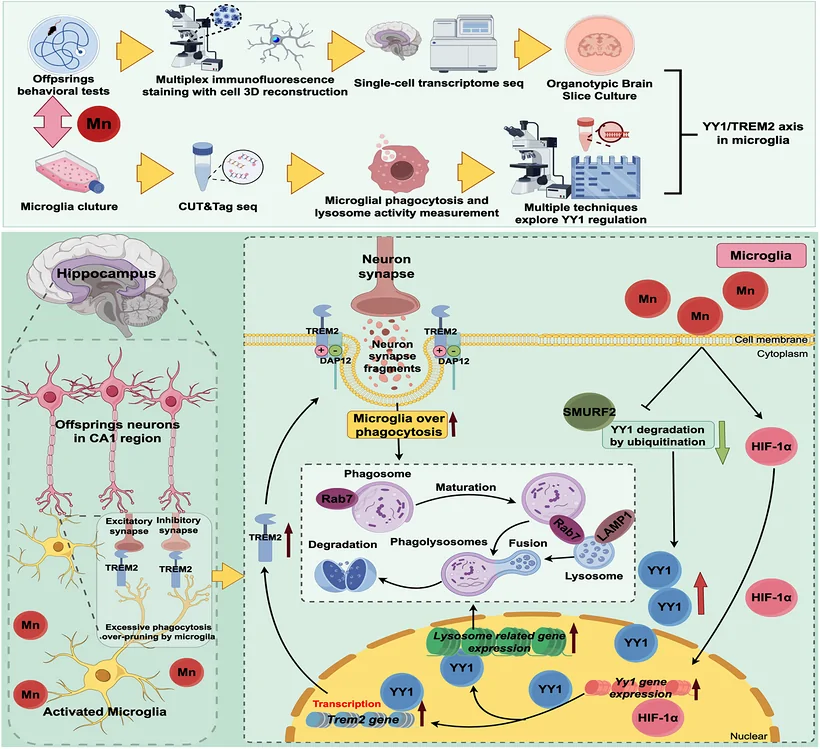

## 1 Introduction

The maturation of the central nervous system during early‐life relies on the coordinated development of neuronal circuits and pruning of synapses. Disruptions during this critical period markedly increase the lifelong risk of neurodevelopmental disorders and learning and memory deficits, which all contribute substantially to the global burden of neurological disease [1, 2, 3]. Among the diverse mechanisms underlying these developmental disturbances, aberrant synaptic remodeling has emerged as a central pathological feature [4, 5]. Synaptic remodeling is particularly important in the hippocampus, where long‐term memory and learning abilities are shaped. However, when this process becomes dysregulated, for example through the excessive elimination of dendritic spines or synapses or the development of imbalances of excitatory and inhibitory synapses, long‐lasting alterations in circuit architecture may arise, ultimately leading to deficits in learning, memory, and behavior [6, 7, 8]. Abnormal synaptic remodeling has been implicated in several neurodevelopmental disorders, including autism spectrum disorders [9], attention‐deficit/hyperactivity disorder (ADHD) [10], and certain forms of cognitive impairment [6].

Microglia serve are key orchestrators of this developmental synaptic remodeling in the hippocampus during early life. Through tightly regulated phagocytosis and activation of phagolysosomal pathways, microglia clear redundant excitatory or inhibitory synapses and sculpt emerging neural networks [11, 12]. Perturbations in microglial phagocytosis can therefore shift the delicate balance between synapse elimination and preservation [13]. In particular, excessive microglial phagocytosis during early life has been shown to cause over‐pruning of synapses and aberrant circuit refinement, thereby contributing to altered learning, memory, and behavioral outcomes [14, 15, 16]. During the activation of microglial phagocytosis, a pathway mediated by triggering receptor expressed on myeloid cells 2 (TREM2) has been recognized as a critical regulator of microglial synaptic clearance [17, 18]. Dysregulated TREM2 activity is associated with synaptic loss and altered microglial behavior across developmental and disease states [19]. Yet despite these insights, the upstream mechanisms governing TREM2 regulation remain unresolved. Yin Yang 1 (YY1), a transcription factor known to participate in microglial activation [20, 21], has recently been implicated in potentially regulating TREM2 expression [22]. However, roles for the YY1/TREM2 axis in microglial phagocytic disorder, which contributes to aberrant synaptic remodeling during development, have not been established.

A growing body of evidence indicates that microglial phagocytic activity is sensitive to a range of environmental factors, which are thus associated with synaptic damage [23, 24, 25]. Among these factors, manganese (Mn) exposure has drawn increasing attention due to its global prevalence and its potential to interfere with early brain development [26]. Early‐life Mn exposure has become a global health concern, especially as epidemiological studies have consistently reported that early‐life Mn exposure is associated with impaired memory and learning performance, reduced attention, and behavioral abnormalities in children [27, 28, 29]. Importantly, the developing brain of neonates and children is particularly vulnerable to Mn exposure, as young offspring exhibit strong gastrointestinal Mn absorption, an immature detoxification capacity, and an incompletely formed blood–brain barrier, leading to increased Mn accumulation during this peak period of synaptic refinement. Recent studies have suggested that Mn may lead to learning and memory deficits by enhancing microglial activation [30, 31], but the relevant molecular mechanisms remain unclear.

In this study, we investigated whether the hippocampal synaptic remodeling induced by early‐life Mn exposure was associated with disorders of microglial phagocytosis when the exposed offspring reached young adulthood. Mn exposure upregulates YY1 levels in developing hippocampal microglia and promotes its direct transcriptional activation of *Trem2* and phagolysosomal pathway‐related genes. These findings show that early‐life Mn exposure disrupts hippocampal synaptic architecture in the developing brain by enhancing microglial engulfment capacity though the YY1/TREM2 axis. By clarifying the role of these proteins in synaptic remodeling, this study suggests new potential therapeutic targets to address early‐life disorders in hippocampal development.

## 2 Results

### 2.1 Early‐Life Manganese Exposure Is Associated with Synaptic Remodeling and Microglial Phagocytosis Activation in Young Adulthood Mice Hippocampus

We established an early‐life Mn exposure mice model from in which pups were continuously exposed to Mn from gestation through lactation (Figure 1A) based on environmental dose of Mn contamination [26, 32, 33]. When the offspring reached young adulthood postnatal day 60 (PND 60), a series of neurobehavioral assays were conducted to assess learning, memory, and anxiety outcomes. To examine potential sex‐specific effects, male and female offspring were analyzed separately.

**FIGURE 1:**
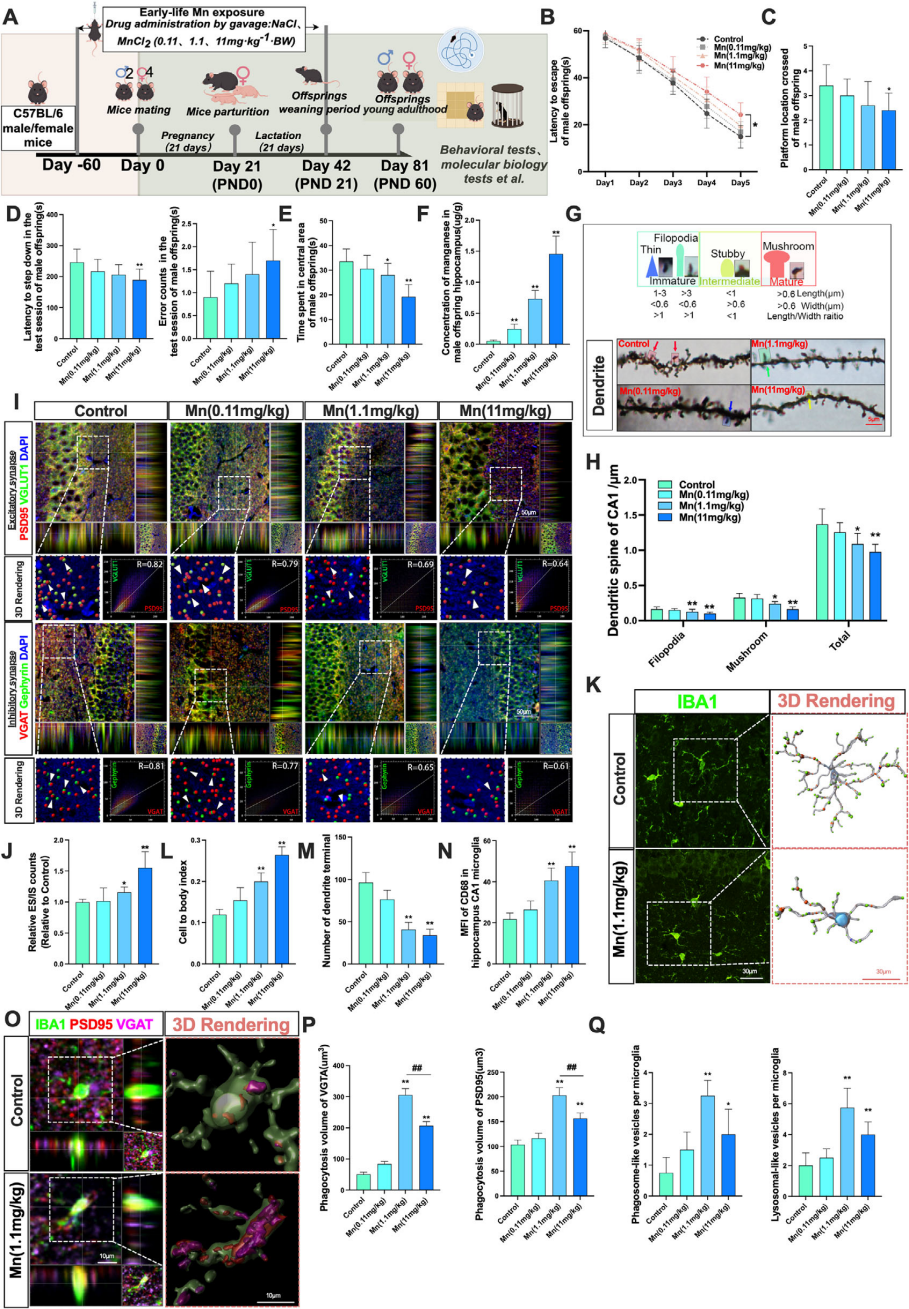
Early‐life Mn exposure induces young adulthood male mice hippocampal synaptic remodeling and activation of microglial phagocytosis. (A) Schematic design of the animal experiment. (B,C) Neurocognitive effects of the positioning navigation and spatial exploration phases in young adulthood male mice Morris water maze. (D) Step‐down test for long time learning and memory ability of young adulthood male mice. (E) Open field test conducted to evaluate young adulthood male mice anxiety and exploratory behavior. (F) Inductively coupled plasma‐mass spectrometry (ICP‐MS) detected manganese accumulation in young adulthood male mice hippocampus. (G,H) Classification for dendritic spines in hippocampus neuron and morphology of enlarged dendritic spines in the CA1 region of the hippocampus, filopodia, mushroom and total spine density (*n* µm^−1^). Scale bar, 5 µm. (I) Representative fluorescence Z‐stack projection images of co‐localized excitatory synaptic markers VGLUT1 (green) and PSD95 (red), and inhibitory synaptic markers VGAT (red) and Gephyrin (green), generated by Imaris 3D reconstruction, with quantitative analysis of Pearson's correlation coefficients. Scale bar, 50 µm. (J) Ratio of excitatory synapses and inhibitory synapses counts relative to Control group. (K) Representative 3D remodeling of microglia (IBA1, green). Scale bar, 30 µm. (L,M) Quantitative analysis of microglial morphological parameters, including cell‐to‐body index and number of dendritic terminals. (N) Quantification of the CD68 mean intensity of fluorescence in IBA1^+^microglia. (O,P) Representative 3D reconstruction of presynaptic and postsynaptic markers PSD95(red) and VGAT (pink) phagocytosis by IBA1^+^ microglia, and the volume of each marker engulfed by microglia within the microscopic field was quantified. Scale bar, 10 µm. (Q) Transmission electron microscopy for identify the number of phagosomes and lysosomes in microglia. Data are presented as mean ± SD. For behavioral assessments and ICP‐MS analysis, *n* = 10 mice per group. For all other in vivo experiments, *n* = 4 mice per group. One‐way analysis of variance (ANOVA) followed by Fisher's LSD post hoc test. ^**^ *p* < 0.05, ^**^ *p* < 0.01 versus Control group; ^##^ *p* < 0.01 versus 1.1 mg kg^−1^ Mn group. See also Figures S1–S3.

Spatial learning and memory were first evaluated in mice exposed to Mn with the Morris water maze. Female mice who had been subjected to the same exposure level during early life showed slight increases in escape latency on the final training day, no other significant changes were observed (Figure S1A–D). By contrast, while male Mn‐exposed mice at 11 mg kg^−1^ displayed marked impairments, including longer escape latencies, fewer platform crossings, and reduced time spent in the target quadrant compared with controls (Figure 1B,C and Figure S1B,D). Long‐term memory was assessed by using the step‐down passive avoidance test. 11 mg kg^−1^ Mn‐exposed male offspring stepped down more quickly and committed more errors during the test session relative to control male mice (Figure 1D), whereas female offspring showed no detectable differences (Figure S1E,F). In the open field test, which evaluates locomotor activity and anxiety‐like behavior, both 1.1 and 11 mg kg^−1^ Mn‐exposed male mice exhibited reduced time spent in the central area, along with decreased total distance traveled and movement speed in 11 mg kg^−1^ Mn exposure male mice (Figure 1E and Figure S1G,I–J). In female mice, Mn exposure was associated with a reduction only in time spent in the central area, with locomotor activity remaining unchanged (Figure S1G–J).

Learning and memory and anxiety‐like behaviors are strongly linked to hippocampal function, particularly within the CA1 subregion, which is essential for emotional regulation, memory integration, and social behavior [34]. Accordingly, we next focused our analysis on the CA1 area. We first certified Mn accumulation in the hippocampus of male offspring exposed to 0.11, 1.1, or 11 mg kg^−1^ Mn and found a clear dose‐dependent increase in Mn levels (Figure 1F). Then, we performed Golgi–Cox staining to evaluate neuronal morphology and dendritic spine structure. Sholl analyses indicated that neuronal arborization was decreased relative to control in male offspring exposed to 1.1 or 11 mg kg^−1^ Mn (Figure S2A,B). Consistent with these structural changes, dendritic spine analyses revealed a significant reduction in total spine density in the hippocampus, including decreases in both immature filopodia and mature mushroom spines, the latter of which are critical for long‐term memory formation (Figure 1G,H). Because synaptic remodeling also involves changes in the balance between excitatory and inhibitory synapses, immunofluorescence staining (Figure 1I) was used identify colocalization of presynaptic and postsynaptic markers of excitatory synapses (presynapse: vesicular glutamate transporter 1, VGLUT1; postsynapse: postsynaptic density protein 95, PSD95) and inhibitory synapses (presynapse: vesicular GABA transporter, VGAT; postsynapse: Gephyrin). Quantification of merged fluorescence intensities and Pearson's correlation coefficients showed reduced excitatory (ES) and inhibitory (IS) synaptic signals in the hippocampus of male offspring exposed to 1.1 or 11 mg kg^−1^ Mn (Figure 1I and Figure S2C,D). To evaluate these changes with greater precision, according to Wang et al. [14], we performed 3D reconstruction of confocal Z‐stack images (0.2 µm step size), which also showed decreased colocalization between presynaptic and postsynaptic markers in the hippocampus male offspring at the 1.1 or 11 mg kg^−1^ exposure levels compared with controls (Figure 1I and Figure S2E,F). We found that the excitatory‐to‐inhibitory synapse (ES/IS) ratio was significantly increased in the hippocampus of these mice (Figure 1J), indicating a synaptic imbalance and supporting the presence of synaptic remodeling at an early‐life exposure level as low as 1.1 mg kg^−1^ Mn.

Because hippocampal synaptic remodeling is strongly driven by microglia‐mediated synaptic pruning and phagocytic activity, we next examined whether microglia were activated. Immunofluorescence staining showed that ionized calcium‐binding adapter molecule 1 (IBA1) positive microglia were prominently distributed throughout the CA1 region (Figure S3A). To rule out the potential influence of peripheral IBA1^+^ monocyte infiltration into the hippocampus induced by early‐life Mn exposure, co‐staining of the microglia‐specific marker transmembrane protein 119 (TMEM119, red) and IBA1 (green) was performed and analyzed with Pearson's correlation coefficients, confirming that this situation was not observed in our model (Figure S3B). Microglial activation was then evaluated using morphological reconstruction and the activation marker CD68. Three‐dimensional reconstruction provided a detailed assessment of microglial morphology (Figure 1K and Figure S3C). Sholl analysis revealed a marked reduction in microglial arbor complexity following Mn exposure (Figure S3D). Consistent with this finding, multiple morphological parameters, including cell‐to‐body index, terminal number, branch number, and total branch length, were significantly decreased in male offspring exposed to 1.1 or 11 mg kg^−1^ Mn, indicating a shift toward an activated, amoeboid‐like phenotype (Figure 1L,M and Figure S3E). In addition, CD68 expression was clearly elevated in CA1 microglia of male offspring at these exposure levels (Figure 1N and Figure S3F), further supporting the presence of microglial activation in response to early‐life Mn exposure.

To evaluate the phagocytic activity of CA1 microglia, tyramide signal amplification (TSA) immunostaining was performed to label IBA1 together with PSD95 and VGAT to mark ES and IS elements, respectively. Three‐dimensional reconstruction of microglia (Figure 1O and Figure S3G) enabled quantitative assessment of engulfed synaptic material. We observed clear increases in microglial uptake of synaptic elements in the hippocampal CA1 regions of 1.1 or 11 mg kg^−1^ Mn‐exposed male offspring, with the higher exposure level correlating with stronger uptake. Notably, though phagocytic activity was increased, male offspring exposed to 11 mg kg^−1^ Mn exhibited a lower engulfment level than did male mice exposed to 1.1 mg kg^−1^ Mn (Figure 1P). Transmission electron microscopy supported these observations by revealing increased numbers of phagosomes and lysosomes in microglia of male offspring exposed to 1.1 or 11 mg kg^−1^ Mn (Figure 1Q and Figure S3H), hinting at activation of the phagolysosomal pathway. Taken together, these findings suggested that early‐life Mn exposure triggers excessive microglial phagocytosis, which contributes to synaptic remodeling in the hippocampal CA1 region of male offspring.

### 2.2 Pharmacological Inhibition of Microglial Activation Reduces Mn‐Induced Synaptic Remodeling and Activation of Microglial Phagocytosis

To further verify whether Mn‐induced microglial phagocytic activation contributes to synaptic remodeling, we used Minocycline (Mino), a widely recognized inhibitor of microglial activation, following the procedures described by Bassett et al. [35] and Yang et al. [36]. We performed these experiments in male mice exposed to the 1.1 mg kg^−1^ dose of Mn, because that was the lowest dose at which we observed detectable microglial activation and synaptic remodeling. The mice were treated with intraperitoneal injections of Mino (10 mg kg^−1^) every three days from PND 6 to PND 21 (Figure 2A). At PND 60, 3D reconstruction showed that Mino treatment markedly restored microglial morphology in the hippocampal CA1 region (Figure 2B). Compared with Mn‐exposed offspring that were treated with vehicle, Mino treatment increased microglial arbor complexity and rescued multiple morphological indices, including the cell‐to‐body index, terminal and branch numbers, and total branch length (Figure 2C–E and Figure S4A,B). CD68 expression in IBA1^+^ microglia was also significantly reduced by Mino treatment (Figure 2F and Figure S4C). As shown in Figure 2G,H, relative to Mn‐exposed male offspring, Mino treatment also decreased the engulfment of synaptic elements. Golgi–Cox staining showed that Mino treatment restored total spine density as well as the abundance of filopodia and mushroom spines in male offspring exposed to 1.1 mg kg^−1^ Mn as compared with 1.1 mg kg^−1^ Mn exposure (Figure 2I,J). Immunofluorescence staining further showed that Mino treatment reversed the 1.1 mg kg^−1^ Mn‐induced reductions in the intensities of fluorescence indicating ES/IS and the Pearson's correlation coefficients (Figure 2K and Figure S4D,E). Three‐dimensional reconstruction of pre‐ and postsynaptic marker colocalization (Figure 2K and Figure S4F,G) revealed that Mino treatment recovered the decreased numbers of ES and IS and alleviated the imbalance of ES/IS ratio induced by early‐life Mn exposure at the 1.1 mg kg^−1^ level (Figure 2L). Together, these findings suggested that pharmacological suppression of microglial phagocytosis mitigates synaptic remodeling in the hippocampal CA1 region of offspring exposed to Mn in the early life.

**FIGURE 2:**
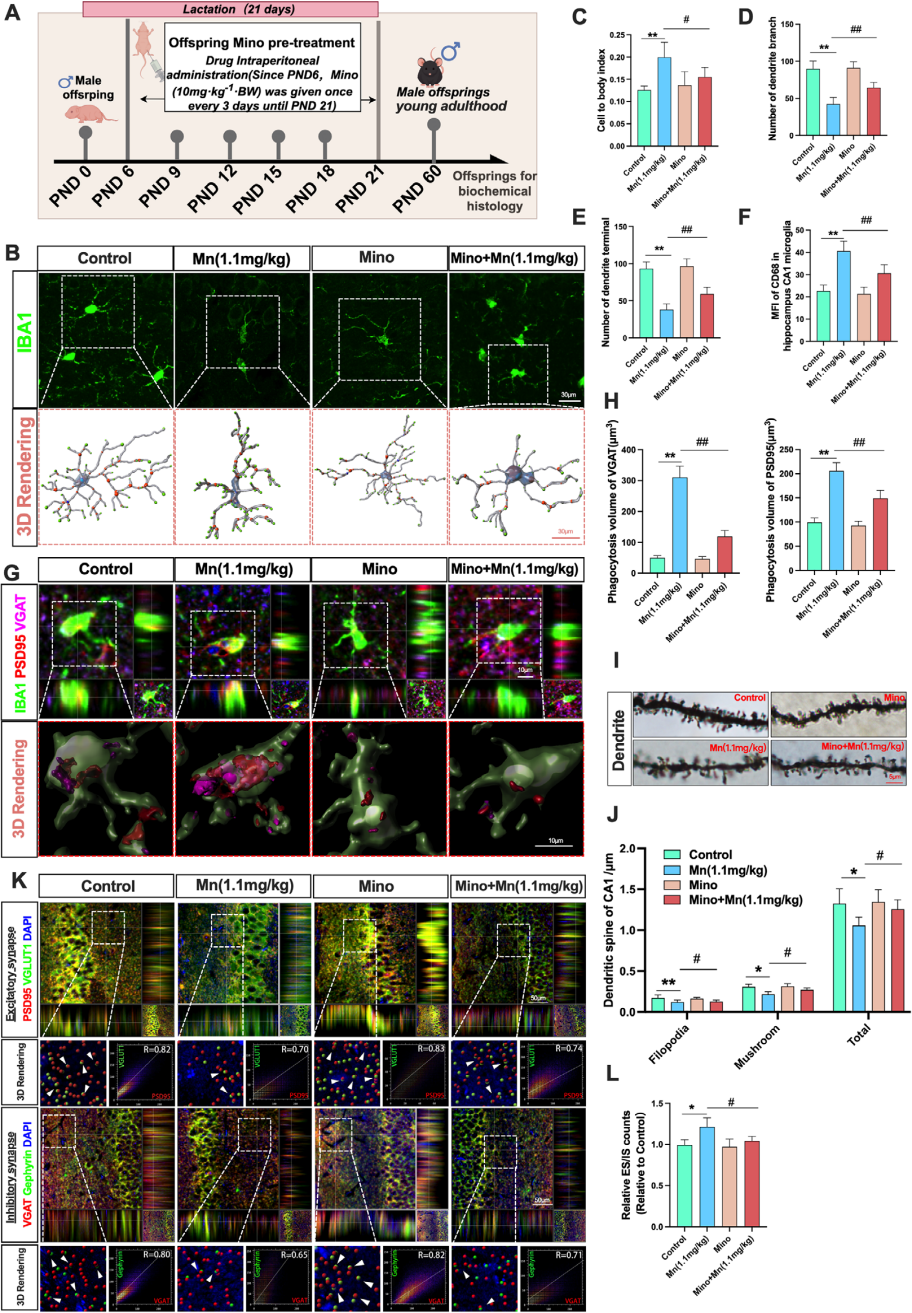
Minocycline attenuates Mn‐induced synaptic remodeling and microglial phagocytic activation. (A) Schematic design map of animal experiment. (B) Representative 3D reconstruction showing microglial (IBA1, green) morphology. Scale bar, 30 µm. (C–E) Quantitative analysis of microglial morphology showing cell‐to‐body index, terminal points, and branch number under different treatment. (F) Quantitative analysis of microglial activation marker CD68 fluorescence intensity within IBA1^+^ microglia. (G,H) Representative immunofluorescence and 3D reconstruction of IBA1^+^ microglia phagocytosis of ES and IS pre‐ or postsynaptic markers (VGAT/PSD95), follow with quantification of phagocytosis levels within the microscopic field. Scale bar, 10 µm. (I,J) Representative Golgi–Cox stained CA1 neuronal dendritic, with quantification of filopodia, mushroom and total spine density (*n* µm^−1^). Scale bar, 5 µm. (K,L) Representative fluorescence Z‐stack projection images of excitatory synaptic markers VGLUT1 (green) and PSD95 (red), and inhibitory synaptic markers VGAT (red) and Gephyrin (green), showing colocalization following Imaris‐based 3D reconstruction, together with Pearson's correlation coefficient analysis and quantification of the ES/IS ratio relative to control. Scale bar, 50 µm. Data are presented as mean ± SD from 4 mice per group. Data were analyzed using two‐way ANOVA followed by Tukey's multiple comparisons test, ^*^ *p* < 0.05, ^**^ *p* < 0.01 versus Control group. ^#^ *p* < 0.05, ^##^ *p* < 0.01 versus 1.1 mg kg^−1^ Mn group. See also Figure S4.

### 2.3 TREM2‐Mediated Microglial Phagocytosis Is Involved in Mn Exposure‐Induced Synaptic Remodeling in the Hippocampus of Male Mice

We performed single‐nucleus RNA sequencing on hippocampal tissues collected from control and 1.1 mg kg^−1^ Mn‐exposed male offspring. After rigorous quality filtering, a total of 68,097 cells were retained for analysis. Using the Seurat R package, we integrated all samples and identified major cell types through t‐distributed stochastic neighbor embedding (t‐SNE) visualization (Figure 3A). Ten distinct clusters were distinguished, including excitatory neurons (ENs), inhibitory neurons (INs), microglia, astrocytes, leptomeningeal cell, fibroblasts, pericytes, oligodendrocytes, and endothelial cell. Notably, male offspring exposed to 1.1 mg kg^−1^ Mn showed an increased ENs/INs ratio compared with controls (Figure 3B), which aligned with our earlier finding of a higher ES/IS ratio in these animals (Figure 1J). Then, we examined microglia heterogeneity and categorized the cells into ten subclusters (MG1–MG10, Figure 3C and Figure S5A). Interestingly we noticed that early‐life Mn exposure led to a significant expansion of the MG1 and MG2 subpopulations, whereas MG3 and MG5 proportions were markedly reduced. To explore the differentiation of microglia subclusters under early‐life Mn exposure, pseudotime trajectory analysis was performed (Figure S5B), and it revealed two major microglial differentiation routes (Fate1 and Fate2) with MG1 and MG2 shifting predominantly toward Fate2 following early‐life Mn exposure. To clarify functional features of MG1 and MG2, we analyzed differentially expressed genes (DEGs) that are upregulated by early‐life Mn exposure. Gene enrichment analyses (Figure S5C,D) showed that DEGs from both the MG1 and MG2 subclusters were enriched in pathways related to phagosomes, lysosomes, and endocytosis. All three of these factors are related to microglia phagocytosis activation, meaning that the pattern of DEGs is consistent with enhanced phagocytic activity during early‐life Mn exposure.

**FIGURE 3:**
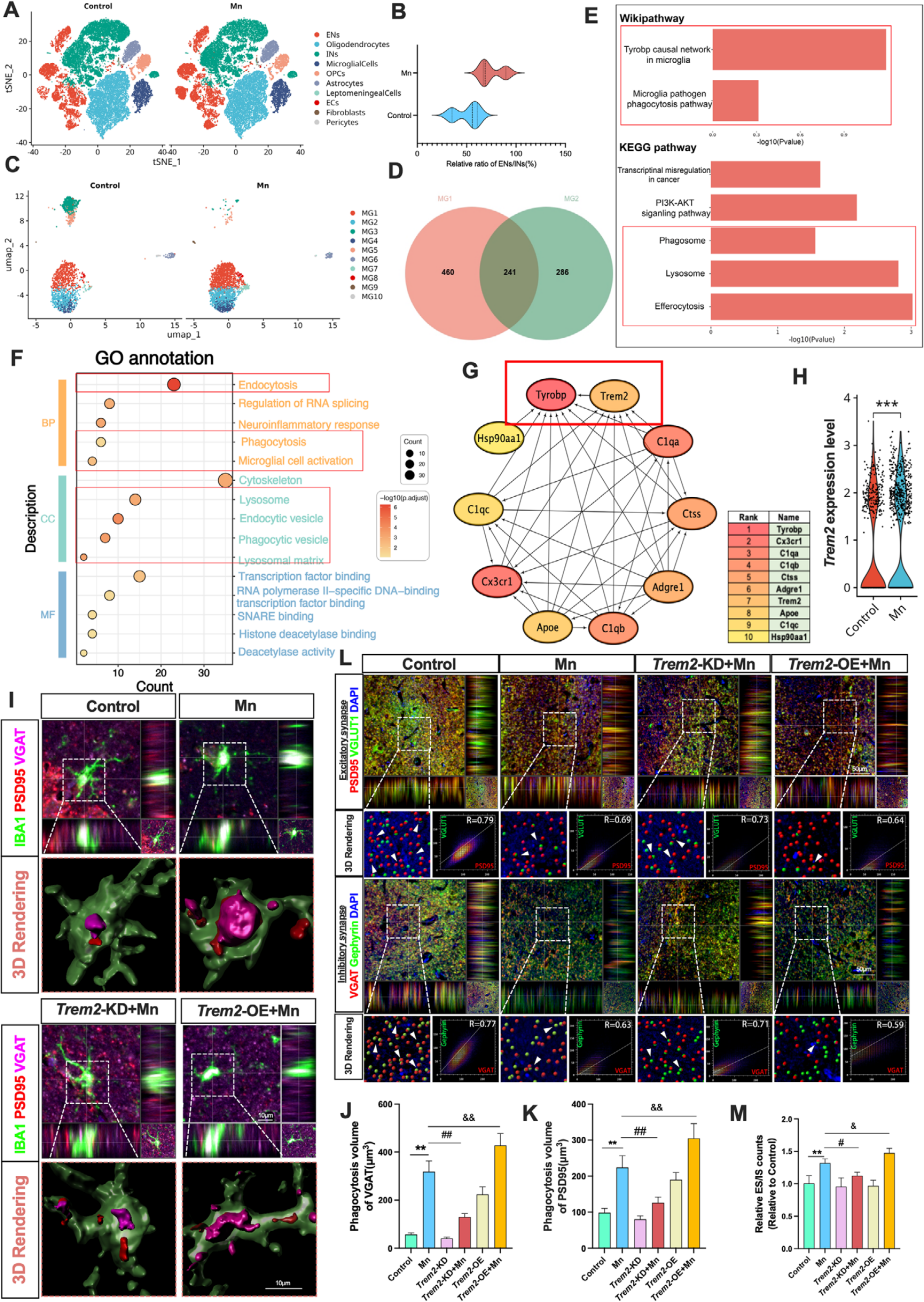
TREM2‐mediated microglial excessive engulfment contributes to synaptic remodeling in the hippocampus of young adulthood male mice exposed to Mn during early life. (A) Visualization of single‐nucleus RNA sequencing transcriptomic profiles in the hippocampus of young adulthood male mice. (B) Relative proportions of excitatory and inhibitory neurons in hippocampal subclusters from Control and Mn in single‐nucleus RNA transcriptomic. (C) Reclustering analysis reveals distinct ten microglia subclusters in Control and Mn exposed offspring. (D) Venn diagram showing the intersection of DEGs between the phagocytic‐featured microglial subclusters MG1 and MG2. (E,F) Visualization of GO, WikiPathway, and KEGG enrichment analyses for the overlapping DEGs between microglia subclusters MG1 and MG2. (G) Cytoscape‐based interaction network of the top 10 hub genes among shared DEGs. (H) Single‐nucleus RNA sequencing analysis of *Trem2* expression in microglia from Control and Mn‐exposed offspring. (I–K) Representative immunofluorescence images and 3D reconstructions showing microglial phagocytosis of pre‐ and postsynaptic proteins under different treatment conditions, along with quantitative analysis of engulfed synaptic proteins within the microscopic field. Scale bar, 10 µm. (L,M) Representative fluorescence Z‐stack projection images showing colocalization of the excitatory synaptic markers VGLUT1 (green) and PSD95 (red), and the inhibitory synaptic markers VGAT (red) and Gephyrin (green) in brain slices, 3D visualization reconstructed by Imaris, along with the corresponding Pearson's correlation coefficients and quantification of ES/IS counts relative to Control. Scale bar, 5 µm. Data are presented as mean ± SD. For snRNA‐seq analysis, *n* = 3 mice per group. For ex vivo brain slice experiments, *n* = 4 biologically independent samples per group. Data were analyzed by two‐way ANOVA followed by Tukey's multiple comparisons test. ^**^ *p* < 0.01, ^***^ *p* < 0.001 versus Control group. ^#^ *p* < 0.05, ^##^ *p* < 0.01 *Trem2*‐KD + Mn versus Mn group. ^&^ *p* < 0.05, ^&&^ *p* < 0.01, *Trem2*‐OE + Mn versus Mn group. See also Figures S5–S7.

To identify the key regulators inducing excessive phagocytosis in abnormally activated microglia, we identified intersecting upregulated in both MG1 and MG2 under Mn exposure and performed Gene Ontology (GO), Kyoto Encyclopedia of Genes and Genomes (KEGG), and WikiPathway analyses (Figure 3D–F). Notably, the common DEGs were enriched in phagocytosis‐associated biological processes and indicated activation of the microglial phagolysosomal pathway. The WikiPathway analysis specifically highlighted a signaling axis involving TREM2, which is encoded by *Trem2*, and its ligand DNAX‐activating protein of 12 kDa (DAP12), which is encoded by *Tyrobp*. Using STRING, we constructed protein–protein interaction (PPI) networks for the intersecting upregulated DEGs (Figure S5E), and subsequent visualization and hub gene identification by Cytoscape highlighted *Trem2* and *Tyrobp* as the central hub (Figure 3G). Consistent with these findings, single‐cell expression profiles showed a robust increase in *Trem2* expression in microglia of 1.1 mg kg^−1^ Mn‐exposed male offspring (Figure 3H).

To validate these observations, we incorporated previously published datasets: one dataset was from a study by Jo et al. [37] that identified DEGs from activated microglia associated with hippocampus‐dependent learning and memory deficits, and the second dataset was from a study by Stratoulias et al. [38] that uncovered microglia prone to activation during early postnatal development. Determination of intersecting DEGs common to these two external datasets and the dataset from the present study (Figure S5F) followed by PPI and key hub identification analyses (Figure S5G,H), again highlighted *Trem2* and *Tyrobp* as the central hub in microglia activation during hippocampus‐associated learning and memory deficits in early postnatal development. Our results also provided evidence confirming elevated TREM2 in offspring exposed to either 1.1 or 11 mg kg^−1^ Mn in the early life (Figure S6A–D). We next examined the activation of microglia phagolysosomal pathway according to our previous enrichment analyses results. Specifically, Ras related protein 7 (RAB7, phagosome marker) [39] and lysosomal‐associated membrane protein 1 (LAMP1, lysosome marker) [40], which were used as markers of phagolysosomal activation, were up‐regulated in 1.1 or 11 mg kg^−1^ Mn‐exposed IBA1^+^ microglia (Figure S6E–G).

We next employed ex vivo brain hippocampal slice cultures to directly test the role of TREM2 in Mn‐induced microglia phagocytic activation and synaptic remodeling. Hippocampal slices were prepared from wild type (WT) C57BL/6 newborn male mice at PND 5 and cultured for 10 day, at which time the slices had become well attached to their substrate and had begun growing from the edges, leading to intact and visible boundaries (Figure S7A). These slices were transduced with adenoviral constructs carrying a microglia‐specific promoter (Figure S7B,C) to generate *Trem2* knockdown (*Trem2*‐KD) or overexpression (*Trem2*‐OE) brain slices. The *Trem2*‐KD and *Trem2*‐OE brain slices transduction efficiency were validated by colocalization of GFP and IBA1^+^ microglia, RT‐qPCR, immunofluorescence staining, and Western blot assays (Figure S7D–H). Suitable conditions for Mn exposure (100 µm Mn for 24 h) were determined using lactate dehydrogenase (LDH) release assays (Figure S7I). Non‐Mn‐exposed *Trem2*‐KD brain slices exhibited significant lower phagocytic capacity toward ES and IS compared to either control or *Trem2*‐OE slices, while phagocytic capacity increased in *Trem2*‐OE slices. Under Mn exposure, *Trem2*‐KD slices exhibited markedly reduced engulfment of synaptic proteins relative to control slices, while Mn‐treated *Trem2*‐OE slices displayed enhanced synaptic uptake (Figure 3I–K and Figure S7N). Golgi–Cox staining revealed no obvious differences in spine morphology among *Trem2*‐KD, *Trem2*‐OE, or control slices ((Figure S7J–M). However, the ratio of relative ES and IS counts was attenuated in Mn‐exposed *Trem2*‐KD slices and exacerbated in Mn‐exposed *Trem2*‐OE slices relative to Mn‐exposed control slices according to immunofluorescence ES/IS 3D‐reconstruction (Figure 3L,M and Figure S7O). Taken together, our results identified TREM2 as a central mediator of the induction of excessive microglial engulfment towards synapses by early‐life Mn exposure, and they revealed the key contribution of TREM2 to synaptic remodeling in the hippocampus.

### 2.4 The Transcription Factor YY1 Regulates TREM2‐Mediated Phagocytosis and Activates the Phagolysosomal Pathway in Microglia

To clarify the molecular mechanisms driving Mn‐induced activation of the phagolysosomal pathway in microglia, we conducted a series of in vitro experiments in SIM A9 microglial cells, which exhibit phenotypic and functional similarities to primary microglia [41] (Figure S8A). According to cell viability assessments, neither 25 nor 50 µm resulted in significant cytotoxicity after 24 h of exposure (Figure S8B); therefore, these concentrations were used in subsequent studies.

Flow cytometry analyses indicated that exposure to 25 or 50 µm Mn triggered microglial activation, as reflected by an increased CD86^+^CD206^−^/CD86^−^CD206^+^ ratio (Figure S8C,D). Expression of the anti‐inflammatory cytokine interleukin‐10 (IL‐10) was increased upon exposure to 25 µm Mn but was suppressed by 50 µm Mn; the levels of pro‐inflammatory cytokines (IL‐6, IL‐1β, TNF‐α) remained unchanged at 25 µm Mn level but were markedly elevated by 50 µm Mn (Figure S8E–L). Phagocytosis assays using fluorescein isothiocyanate (FITC)‐labeled latex beads were performed in order to evaluate Mn‐induced phagocytic activation according to Wang et al. [42]. Microglia exposed to either 25 or 50 µm Mn showed enhanced phagocytic capacity, with 3D‐reconstruction demonstrating increased bead volume and overall engulfment rate (Figure 4A–C and Figure S8M). However, phagocytic efficiency at 50 µm Mn level was reduced relative to that at 25 µm, indicating a phagocytic functional decline at the higher concentration. To comprehensively assess lysosomal function, we evaluated lysosomal abundance, acidification, and acidic vesicular content by LysoTracker‐Red, LysoSensorYellow/Blue, and acridine orange staining, respectively. The results consistently showed that 25 µm Mn exposure markedly enhanced microglia lysosomal function, whereas 50 µm Mn treatment resulted in a decline relative to 25 µm Mn treatment (Figure 4D–F and Figure S8N,O). Increased colocalization of RAB7 and LAMP1 was observed in microglia exposed to 25 or 50 µm Mn, indicating phagolysosomal activation; however, colocalization was diminished at the 50 µm Mn level compared with that at 25 µm (Figure 4G and Figure S8P). Together, these findings indicated that while Mn exposure does not impair microglial viability at doses of 25 or 50 µm, exposure to 25 µm Mn led to optimal phagolysosomal activation.

**FIGURE 4:**
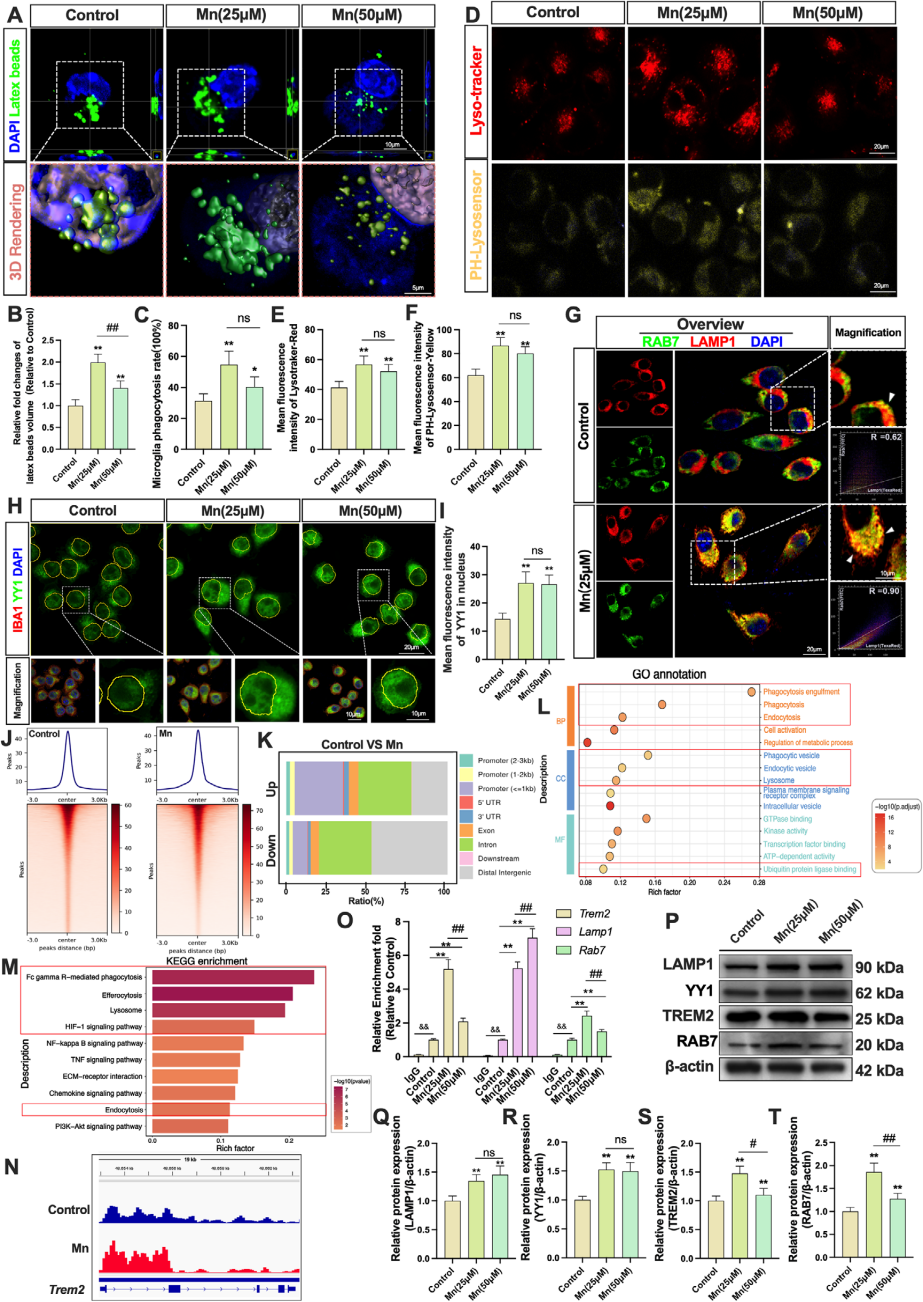
YY1 transcriptionally regulates TREM2 and activation of the microglial phagolysosomal pathway. (A) Representative immunofluorescence and 3D reconstruction images showing microglia phagocytosis of latex beads under different Mn exposure concentrations. Scale bar, 10 µm, magnification 3D model scale bar, 5 µm. (B,C) Quantification of fold changes in microglial phagocytic volume relative to the Control group and the proportion of phagocytic microglia within the microscopic field. (D–F) Representative fluorescence images and quantitative analysis of LysoTracker‐Red and PH‐LysoSensor Yellow/Blue signals in microglia under different Mn exposure. Scale bar, 20 µm. (G) Representative fluorescence images showing activation of the phagocytic (RAB7, green) lysosomal (LAMP1, red) pathway in microglia under different Mn exposure, with Pearson's colocalization coefficients. Scale bar, 20 µm, magnification scale bar, 10 µm. (H,I) Representative fluorescence images and quantitative analysis of nuclear YY1 (green) expression in IBA1^+^(red) microglia under different Mn exposure concentrations. Scale bar, 20 µm, magnification scale bar, 10 µm. (J) Heatmap and quantitative analysis of signal intensity for each YY1 CUT&Tag sequencing peak in Control and Mn (25 µm) groups. (K) Distribution of YY1 CUT&Tag sequencing peaks relative to transcription start sites in control and Mn (25 µm) groups. (L,M) GO and KEGG enrichment analyses of DEGs identified from YY1 CUT&Tag sequencing. (N) CUT&Tag results showed YY1 binding sites in gene *Trem2*. (O) Quantitative CUT&Tag qPCR results showing the relative enrichment of YY1 at *Trem2* and phagolysosomal pathway (*Rab7* and *Lamp1*) with various Mn exposure, relative to Control. (P–T) Western blot analysis of YY1, TREM2, and phagolysosomal pathway (LAMP1 and RAB7) in microglia under different Mn exposure concentrations, protein expression levels normalized to β‐actin and relative to Control. Data were expressed as mean ± SD. Sample sizes *n* = 3 independent experiments for Western blot analysis, and *n* = 4 independent experiments for other assays. One‐way ANOVA followed by Fisher's LSD post hoc test. ^*^ *p* < 0.05, ^**^ *p* < 0.01 versus Control group. ^#^ *p* < 0.05, ^# #^ *p* < 0.01 versus Mn (25 µm) group. ^&&^ *p* < 0.01 versus lgG group. See also Figures S8 and S9.

Single‐cell regulatory network inference and clustering (SCENIC) single‐cell transcription profiles (Figure S9A–C) indicated that YY1 may serve as an upstream transcriptional regulator of Mn‐induced phagocytic microglial subpopulations. Functional analysis of Mn‐induced DEGs with putative YY1 binding sites revealed enrichment in Mn^2^^+^ transport, phagosome maturation, and other phagocytosis‐related processes (Figure S9D,E), suggesting a potential regulatory role for YY1 in the TREM2‐mediated phagocytosis and phagolysosomal activation. Database analyses using UCSC, JASPAR, and GEPIA identified putative YY1 binding motifs within the *Trem2* promoter and demonstrated strong co‐expression of *Trem2* and *Yy1* in hippocampus (Figure S9F,G).

To verify our YY1 role during Mn exposure, immunofluorescence was firstly performed and confirmed that Mn exposure markedly promoted expression of YY1 in microglia nuclear, and in similar levels in microglia at the 25 or 50 µm level (Figure 4H,I). To continue provide direct evidence for YY1‐mediated transcriptional regulation of phagolysosomal processes, cleavage under targets and tagmentation (CUT&Tag) sequencing was performed in control‐treated or 25 µm Mn‐exposed microglia, and prominent YY1 nuclear enrichment and strong peak signals were obtained (Figure 4J,K). Differential peak analyses identified YY1‐binding genes that are differentially expressed under 25 µm Mn exposure (Figure S9H) and GO/KEGG enrichment revealed a significant association of these DEGs with phagolysosomal pathways (Figure 4L,M). Visualization of CUT&Tag peaks demonstrated robust YY1 binding at *Trem2*, *Rab7*, and *Lamp1* loci under 25 µm Mn exposure (Figure 4N and Figure S9I). Following CUT&Tag‐qPCR confirmed that exposure to 25 µm Mn increased YY1 occupancy at these loci; interestingly, 50 µm Mn exposure led to a lower YY1 binding capacity compared to 25 µm Mn (Figure 4O). Taken together, the results suggested that Mn exposure induces YY1 to promote transcription of *Trem2*, *Rab7*, and *Lamp1*. Consistent with these findings, RT‐qPCR and Western blot analyses showed that exposure to 25 µm Mn markedly increased microglial YY1 expression and upregulated the levels of TREM2, RAB7, and LAMP1, whereas exposure to 50 µm Mn was associated with smaller induction of expression of TREM2, RAB7, and LAMP1 (Figure 4P–T and Figure S9J). Collectively, these results indicated that Mn exposure enhanced expression of YY1 in microglia and its nuclear, leading to the promotion of transcription of *Trem2* and key phagolysosomal genes.

### 2.5 Downregulation of *Yy1* Attenuates the Excessive Activation of the Phagolysosomal Pathway in Mn‐Exposed Microglia

To further investigate the role of YY1 in increasing TREM2‐mediated phagocytosis and activation of the phagolysosomal pathway under Mn exposure, we performed a genetic knockdown of the expression of *Yy1* in SIM A9 cells (*Yy1*‐KD; Figure S10A–D) and exposed these cells to 25 µm Mn. The up‐regulated YY1 expression in microglia nuclear and enhanced occupancy of YY1 at the *Trem2* promoter induced by 25 µm Mn exposure was alleviated in *Yy1*‐KD microglia (Figure 5A–D and Figure S10E). We next explored whether loss of *Yy1* in microglia affected TREM2‐mediated phagocytic capacity and phagolysosomal pathway activation. FITC latex bead engulfment assays revealed that *Yy1*‐KD microglia exhibited significantly diminished phagocytic capacity compared to controls. And 25 µm Mn treatment cannot induced the phagocytic capacity activation of *Yy1*‐KD microglia which was markedly reduced in comparison with that of control and Mn‐exposed microglia (Figure 5E–G). *Yy1*‐KD microglia had lower lysosomal function in comparison with control cells in the absence of Mn exposure or not. Similarly, in the context of exposure to 25 µm Mn‐treated, *Yy1*‐KD cells displayed notable reductions in lysosomal abundance, acidification, and overall lysosomal activation relative to control and Mn‐exposed cells (Figure S10F–J). Moreover, *Yy1*‐KD microglia showed markedly reduced RAB7–LAMP1 colocalization following 25 µm Mn exposure compared with those of control microglia under 25 µm Mn exposure, indicating that downregulation of *Yy1* suppressed Mn‐induced phagolysosomal activation (Figure S10K). *Yy1*‐KD attenuated the upregulation of expression of phagolysosome‐associated genes (*Trem2*, *Rab7*, and *Lamp1*) that is induced by exposure to 25 µm Mn (Figure 5H). *Yy1*‐KD microglia displayed reductions in Mn‐induced increased expression of TREM2, RAB7, and LAMP1 (Figure 5I–L).

**FIGURE 5:**
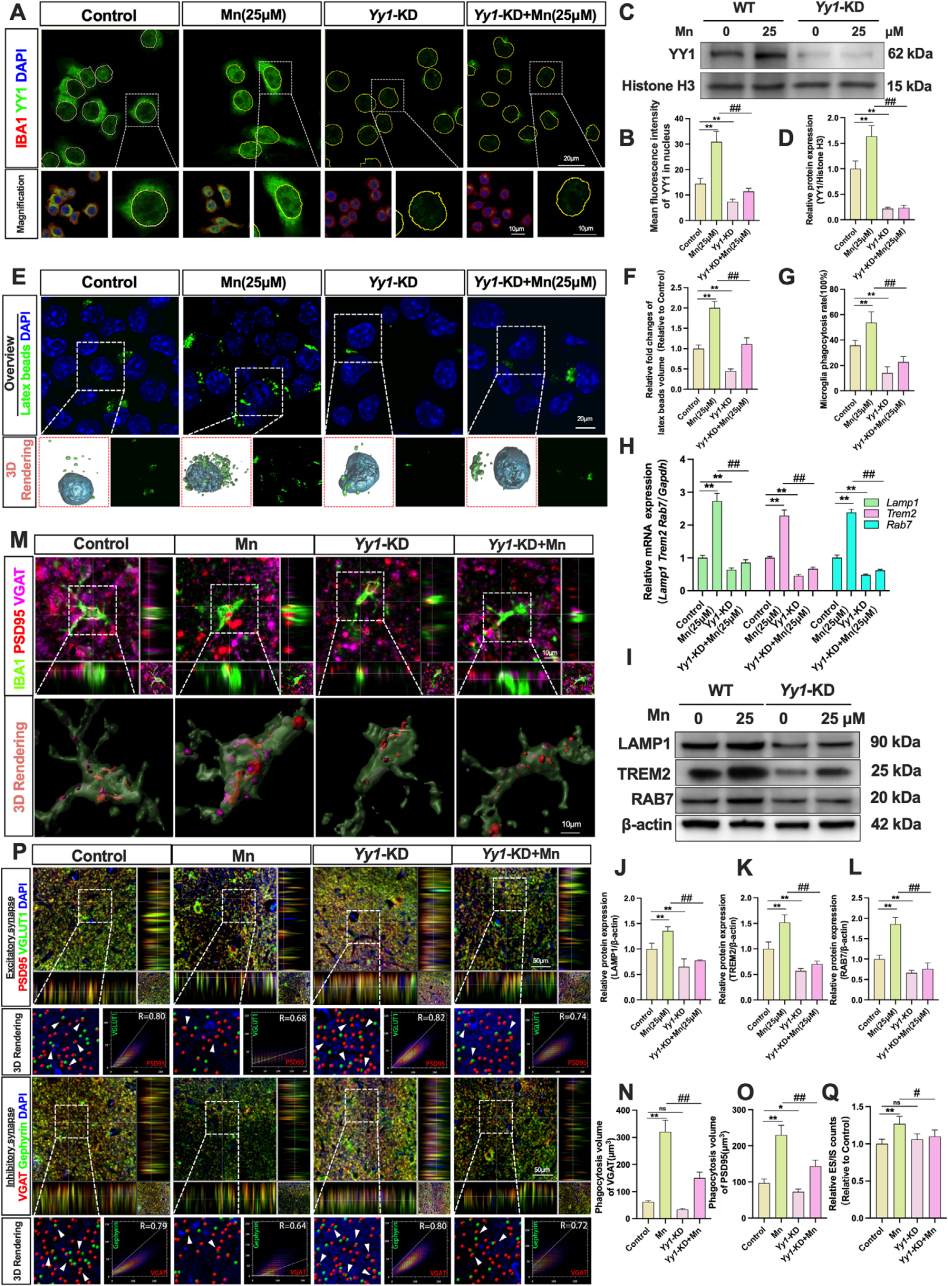
Suppression of *Yy1* alleviates the excessive of TREM2‐mediated phagocytosis and activation of the phagolysosomal pathway in Mn‐exposed microglia. (A,B) Representative immunofluorescence staining and quantification of YY1 nuclear localization in IBA1^+^ (red) microglia across different treatment groups. Scale bar, 20 µm, magnification scale bar,10 µm. (C,D) Western blot showing YY1 expression in nuclear and quantification. (E–G) Representative images and 3D reconstructions of latex bead phagocytosis in microglia from various treatment group, with quantitative analysis of phagocytic capacity (relative to Control) and phagocytic microglia rate (%) within the microscopic field. Scale bar, 20 µm. (H) mRNA expression of *Trem2* and phagolysosomal pathway–related genes (*Rab7* and *Lamp1*) in different treatment groups determined by RT‐qPCR. (I–L) Western blot analysis showing relative protein expression levels of TREM2 and phagolysosomal pathway (RAB7 and LAMP1) in microglia under different treatments. (M–O) Representative 3D reconstruction images of IBA1^+^ microglia within the microscopic field illustrating engulfment of PSD95 and VGAT, along with quantification of phagocytic volume. Scale bar, 10 µm. (P,Q) Representative fluorescence Z‐stack projection images reconstructed using Imaris 3D software, showing colocalization of VGLUT1 (green) and PSD95 (red), and VGAT (red) and Gephyrin (green), along with Pearson's correlation coefficient analysis and quantification of ES/IS counts relative to Control. Scale bar, 50 µm. mRNA expressions were normalized to *Gapdh* and relative to Control, proteins expressions were normalized to Histone H3 or β‐actin and relative to Control. Data were expressed as mean ± SD. For Western blot analysis, *n* = 3 independent experiments; and for ex vivo brain slice and other experiments, *n* = 4 independent samples per group. Data were analyzed by two‐way ANOVA followed by Tukey's multiple comparisons test. ^*^ *p* < 0.05, ^**^ *p* < 0.01 versus Control group. ^#^ *p* < 0.05, ^# #^ *p* < 0.01 versus Mn group. See also Figures S10 and S11.

Further experiments using the ex vivo brain slice model to establish *Yy1* knockdown (*Yy1*‐KD) in microglia were performed (Figure S11A–E), and Mn exposure condition were the same as the ex vivo brain slice model describe above (100 µm Mn, 24 h). We observed that TREM2 expression in *Yy1*‐KD slices was significantly reduced compared with control slices, and more importantly, *Yy1* knockdown markedly alleviated the Mn‐induced increase in TREM2 expression observed in WT slices, providing further evidence that YY1 regulates TREM2 expression in microglia (Figure S11F,G). To further evaluate microglial phagocytosis changes, we assessed synaptic markers (VGAT and PSD95) engulfed by microglia in WT and *Yy1*‐KD brain slices. Quantification of microglial engulfment volume of PSD95 and VGAT showed that, compared with WT controls, *Yy1*‐KD slices exhibited reduced phagocytic volume (Figure 5M–O). In parallel, we used the same analytical strategy described previously to evaluate excitatory and inhibitory synaptic marker colocation and relative balance in brain slices. We found that *Yy1* knockdown alleviated the Mn‐induced reduction colocation of excitatory and inhibitory synaptic markers, as well as the imbalance in excitatory/inhibitory synaptic ratio (Figure 5P,Q and Figure S11H,I). Together, these works provide evidence for the mechanism that the YY1/TREM2 axis mediates excessive microglial phagocytosis and activation of the phagolysosomal pathway which related to abnormal synaptic remodeling under Mn exposure.

### 2.6 Upregulation of YY1 Involves both HIF1α Activity and Dysfunction of Ubiquitin‐Mediated Degradation

Based on our previous sequencing data, enrichment analyses were performed. These analyses suggested that Mn exposure activated the HIF1 signaling pathway and disrupted the ubiquitin–proteasome degradation machinery (Figure 4L,M), providing new mechanistic insight that both pathways may contribute to elevated YY1 levels. Given previous reports that Mn activates members of the HIF1 family [43], we first examined whether the HIF1 pathway regulated transcription of the *Yy1* gene. By screening JASPAR and GEPIA databases, we identified hypoxia inducible factor 1α (HIF1α) as the most likely transcriptional regulator of YY1. Specifically, JASPAR predicted a putative HIF1α binding motif within the *Yy1* promoter (Figure 6A), and GEPIA revealed a positive co‐expression relationship between *Hif1a* and *Yy1* in hippocampal tissue (Figure 6B). To validate the importance of this regulatory axis under Mn exposure, we generated *Hif1a*‐knockdown (*Hif1a*‐KD) microglial cells (Figure S12A–D). Chromatin immunoprecipitation (ChIP) assays confirmed the direct binding of HIF1α to the *Yy1* promoter (Figure 6C), establishing HIF1α as a transcriptional activator of *Yy1*, and this binding was markedly enhanced following 25 µm Mn exposure. To exclude the possibility that HIF1α directly regulates *Trem2* under 25 µm Mn exposure, we further examined the binding of HIF1α to the *Trem2* promoter. No significant difference in HIF1α occupancy at the *Trem2* promoter was observed between the control and Mn‐treated groups (Figure S12E), indicating that the Mn‐induced upregulation of *Trem2* is unlikely to result from direct transcriptional regulation by HIF1α. Immunofluorescence and nuclear protein analyses revealed a marked attenuation of Mn‐induced YY1 nuclear accumulation in *Hif1a*‐KD cells compared to control microglia (Figure 6D–G). Knockdown of *Hif1a* expression significantly reduced the Mn‐induced increases in mRNA and protein levels of TREM2, RAB7, and LAMP1 (Figure 6H–M). Collectively, these findings demonstrated that HIF1α directly binds to the *Yy1* promoter, enhancing *Yy1* transcription and activation of the YY1/TREM2‐mediated phagolysosomal pathway under Mn exposure.

**FIGURE 6:**
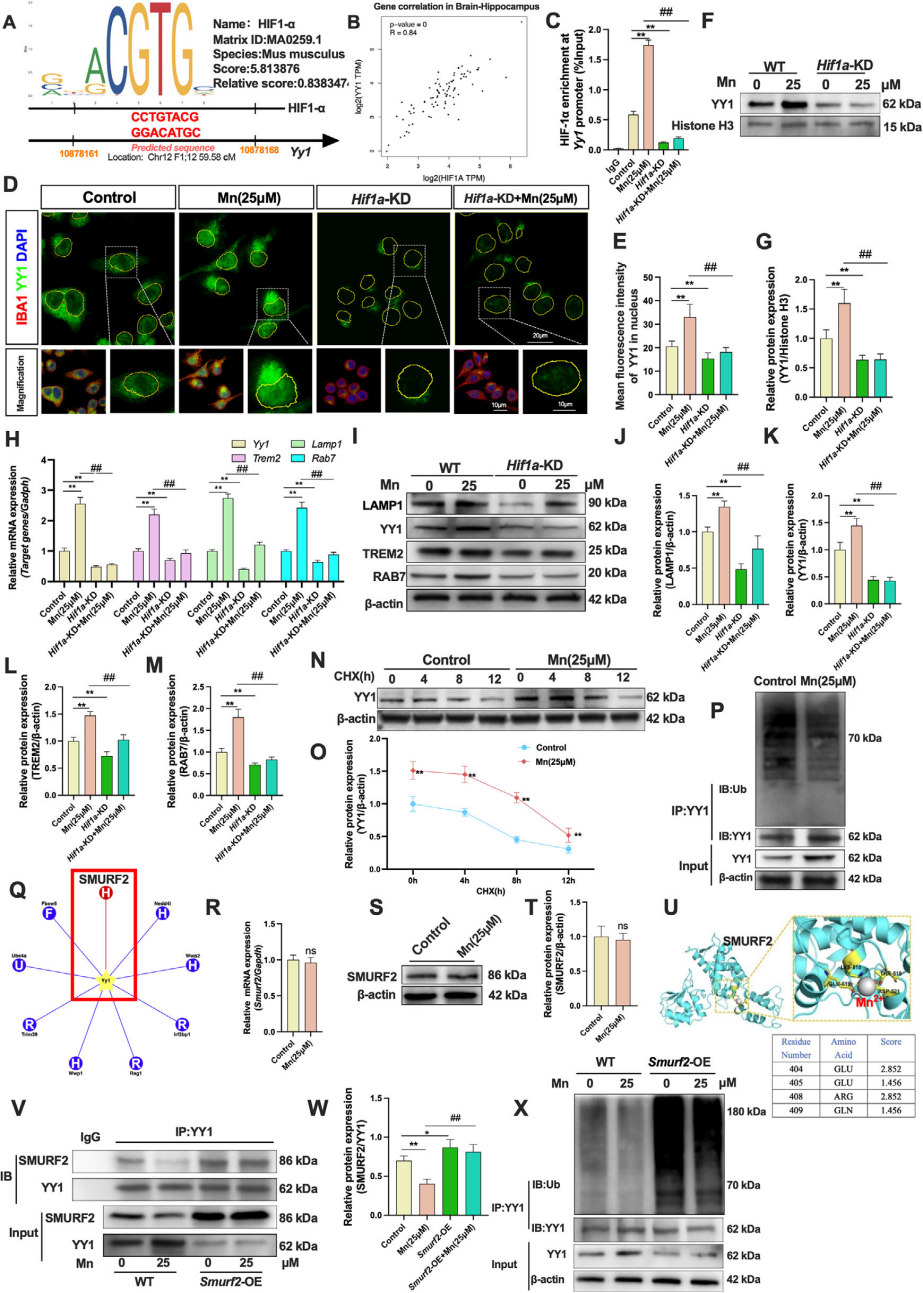
HIF1α regulation and dysfunction of SMURF2‐mediated ubiquitin degradation are responsible for YY1 upregulation. (A) Predicted HIF1α binding sites in the *Yy1* promoter region from the JASPAR database. (B) Co‐expression analysis of *Hif1a* and *Yy1* in the hippocampus by GEPIA database. (C) ChIP assay showing the binding between HIF1α and the *Yy1* promoter. (D,E) Representative immunofluorescence images and quantitative analysis showing the nuclear expression of YY1 under different treatments. Scale bar, 20 µm, magnification scale bar, 10 µm. (F,G) Western blot analysis of nuclear YY1 protein levels in microglia from different treatment groups. (H) RT‐qPCR analysis of *Yy1*, *Trem2*, and phagolysosomal pathway‐related genes (*Rab7* and *Lamp1*) under various treatments. (I–M) Western blot and quantification of YY1, TREM2, and phagolysosome (RAB7 and LAMP1) proteins under different treatments. (N,O) Western blot showing YY1 protein degradation dynamics following CHX treatment at different time points under Mn exposure. (P) Immunoprecipitation analysis of YY1 ubiquitination levels in microglia. (Q) Identification of the E3 ubiquitin ligase SMURF2 targeting YY1 by the UbiBrowser 2.0 database. (R) Relative mRNA expression level of *Smurf2* in Mn treatment assessed by RT‐qPCR. (S,T) Western blot and quantification of SMURF2 relative protein expression level under Mn treatment. (U) Molecular docking prediction of potential Mn^2^^+^ binding sites within SMURF2. (V,W) Co‐immunoprecipitation assays revealing the interaction strength between SMURF2 and YY1 under different treatments. (X) Analysis of YY1 ubiquitination levels after immunoprecipitation across different experimental groups. mRNA expressions were normalized to *Gapdh* and relative to Control, proteins expressions were normalized to Histone H3 or β‐actin and relative to Control. Data were expressed as mean ± SD. For in vitro experiments, *n* = 3 independent experiments for Western blot analysis, and *n* = 4 independent experiments for all other assays. Data were analyzed by two‐way ANOVA followed by Tukey's multiple comparisons test. ^*^ *p* < 0.05, ^**^ *p* < 0.01 versus Control group. ^##^ *p* < 0.01 versus Mn (25 µm) group. See also Figure S12.

Upon application of the protein synthesis inhibitor cycloheximide (CHX), we observed that Mn treatment markedly enhanced the stability of YY1 protein (Figure 6N,O). Immunoprecipitation analyses also revealed a pronounced reduction in YY1 ubiquitination following Mn exposure (Figure 6P), suggesting impaired ubiquitin‐dependent degradation. Because ubiquitination‐mediated degradation is typically controlled by E3 ubiquitin ligases, we queried the UbiBrowser 2.0 database and identified SMAD specific E3 ubiquitin protein ligase 2 (SMURF2) as a potential E3 ligase for YY1 (Figure 6Q). We then generated a *Smurf2*‐overexpressing (*Smurf2*‐OE) microglial cell (Figure S12A,F–H) to test this prediction. Co‐IP and IP assays confirmed a direct interaction between SMURF2 and YY1 (Figure S12I,J). Treatment of control cells with the proteasome inhibitor MG132 led to YY1 accumulation while *Smurf2*‐OE microglia attenuated this effect of MG132 (Figure S12K,L). CHX‐based assay further demonstrated reduced YY1 stability in *Smurf2*‐OE microglia (Figure S12M,N). These results collectively confirmed that SMURF2 mediates YY1 ubiquitin‐dependent degradation. However, there are no changes observed in mRNA and protein levels of SMURF2 between control and 25 µm Mn‐treated microglia (Figure 6R–T), ruling out the induction of transcriptional or translational alterations to SMURF2 levels by Mn. But following molecular docking analyses predicted multiple sites within SMURF2 where Mn^2^^+^ might bind, thus potentially altering its conformation and impairing its interaction with YY1 (Figure 6U). Supporting this hypothesis, Co‐IP assays demonstrated that Mn exposure weakened SMURF2–YY1 binding in control SIM A9 microglia and in *Smurf2*‐OE cells (Figure 6V,W). IP analysis showed reduced YY1 ubiquitination in control or *Smurf2*‐OE cells following Mn treatment (Figure 6X). Together, these findings revealed that Mn exposure interferes with SMURF2 function, diminishes its interaction with YY1, and suppresses SMURF2‐mediated ubiquitin‐dependent degradation, ultimately leading to enhanced stabilization and accumulation of YY1.

## 3 Discussion

Environmental Mn exposure during early life remains a widespread yet underappreciated public health concern, particularly in regions where environmental Mn accumulates in soil, water, or food chains [44, 45, 46]. Recent epidemiological findings suggest that environmentally relevant Mn exposure during critical windows of early‐life development may exert neurodevelopmental toxicity [47, 48, 49]. However, experimental evidence from in vivo models and mechanistic insights into the molecular pathways underlying these effects remain limited. In the present study, environmentally relevant Mn exposure levels were defined based on epidemiological data and employed to establish an animal model that mimics real‐world early‐life Mn exposure. In our work, we found that early‐life environmental Mn exposure led to impairments in learning, long‐term memory, and occurrence of anxiety‐related behaviors in young adulthood mice males. These behavioral alterations were accompanied by pronounced synaptic remodeling in the hippocampus. Mechanistically, aberrant activation of microglia and excessive engulfment contributed to Mn‐induced synaptic remodeling. Furthermore, we identified a YY1‐dependent regulatory axis that transcriptionally activates TREM2 and the phagolysosomal pathway, thereby driving excessive microglial synaptic engulfment.

Chronic environmental Mn exposure is associated with ADHD [49] and cognitive and memory impairments in young adulthood males [50]. Accordingly, we selected PND 60, corresponding to young adulthood in mice, as the time point to conduct neurobehavioral tests and demonstrated that early‐life exposure to environmentally relevant Mn doses resulted in impairments in learning ability, long‐term memory, and elevated anxiety‐like behaviors. Notably, these behavioral alterations exhibited a clear sex dependence. Consistent with epidemiological observations, male offspring displayed more pronounced behavioral deficits than did female offspring. Although the mechanistic basis for this sex difference remains unclear, early‐life developmental timing, hormonal milieu, and microglial maturation trajectories may contribute to divergent susceptibility [51, 52]. Moreover, recent single‐nucleus RNA sequencing studies have revealed pronounced sex‐specific heterogeneity among activated microglial subpopulations, highlighting that male and female microglia may exhibit distinct transcriptional and functional responses under pathological conditions [53]. Given that the present study identified enhanced phagocytic activation of microglia as a key mechanism underlying Mn‐induced synaptic remodeling, it is possible that sex‐dependent differences in specific microglial subpopulations, particularly phagocytosis‐related subsets, may contribute to the greater behavioral susceptibility observed in male offspring following early‐life Mn exposure. These findings provide a potential mechanistic explanation for the sex‐dependent neurobehavioral effects of Mn, although further studies are needed to characterize sex‐specific microglial subtypes.

The behavioral abnormalities and deficits in learning and memory observed in male mice following early‐life environmental Mn exposure were closely associated with hippocampal dysfunction. The hippocampal CA1 region is a critical brain area involved in spatial learning, long‐term memory consolidation, social behavior, and emotional regulation, and it is particularly sensitive to Mn‐induced neurotoxicity [54] and prone to abnormal synaptic remodeling [55, 56]. We found aberrant elevation of the ES/IS ratio and synaptic remodeling might be driven by excessive microglial phagocytic activation during early‐life Mn exposure. Notably, despite more severe abnormal synaptic remodeling and higher overall microglial activation observed in mice who had been exposed to an Mn dose of 11 mg kg^−1^ as compared with a dose of 1.1 mg kg^−1^, microglial phagocytic activity was attenuated in the context of the higher dose. This interesting phenomenon may reflect direct neurotoxic or synaptotoxic effects of higher Mn doses on neurons and synapses, or a dose‐dependent phenotypic shift of microglia from a phagocytically active state toward a more inflammation‐associated state.

To specifically dissect the contribution of microglial phagocytic dysfunction to the effects of Mn, we selected 1.1 mg kg^−1^ Mn as the exposure dose for subsequent in vivo experiments focusing on the relationship between excessive microglial phagocytosis and abnormal synaptic remodeling. Based on previous reports [13, 36], we pharmacologically inhibited microglial activation in offspring mice using Mino, which was administered after birth. Our findings demonstrated that early‐life environmentally relevant Mn exposure promotes aberrant microglial phagocytic activation, leading to excessive synaptic engulfment and subsequent hippocampal abnormal synaptic remodeling.

To further elucidate the mechanisms underlying this phenomenon, we performed hippocampal single‐nucleus RNA sequencing in male mice from the control or 1.1 mg kg^−1^ Mn exposure groups. Notably, two phagocytosis‐enriched subpopulations, designated MG1 and MG2, were markedly expanded under early‐life environmental Mn exposure. Differential gene expression and pathway enrichment analyses confirmed that these subpopulations exhibit prominent phagocytic signatures, accompanied by robust activation of the phagolysosomal pathway, supporting their functional role in synaptic engulfment. We constructed co‐expression networks and identified TREM2 as a central regulatory node. Cross‐validation using previously published datasets [37, 38] supported a pivotal role for TREM2 in mediating microglial‐driven hippocampal learning and memory impairment in Mn‐exposed mice. Given the well‐established role of TREM2 in lipid‐sensing and lipid‐associated phagocytosis [57], we proposed that TREM2 represents the key molecular mediator driving microglial phagocytic activation. Moreover, knockdown of *Trem2* in hippocampal slices exhibits reduced microglial phagocytic activity and alterations in the ES/IS ratio, while overexpression of *Trem2* in hippocampal slices shows the opposites. However, interestingly, Golgi–Cox staining results showed no detectable changes in dendritic spine indices between *Trem2*‐KD and *Trem2*‐OE slices. This may be due to the relatively short Mn exposure window following adeno‐associated viral transfection in microglia compared with the prolonged exposure that occurs during early development in situ, indicating that feasible structural remodeling may require longer perturbation than those performed on slice cultures, and it may also suggest that the window for developing microglial phenotypic changes is narrow.

Next, we conducted a series of in vitro mechanistic investigations to clarify enhanced phagocytic activity and activation of the phagolysosomal pathway. Our results revealed 25 µm Mn treatment represented a functional peak of microglial phagocytic and phagolysosomal activation, whereas 50 µm Mn treatment partly decreased phagocytosis phagolysosomal activity and up‐regulates inflammation. However, during this process, we found an increase of CD206^+^CD86^+^ double‐positive microglia under Mn treatment, suggests the emergence of a transitional activation state rather than a classical binary M1/M2 polarization. In the context of Mn exposure, microglia may undergo functional reprogramming characterized by simultaneous upregulation of pro‐inflammatory (CD86) and repair‐associated (CD206) markers. This mixed phenotype is consistent with a disease‐associated microglial state [58], in which inflammatory signaling coexists with changed phagocytic and tissue remodeling capacity [59, 60]. Such a phenotype may reflect an intermediate stage of microglial activation driven by TREM2 mediated metabolic and epigenetic remodeling under Mn‐induced stress. Following reanalysis of single‐cell transcriptional sequencing led to our identification of YY1 as a potential transcriptional regulator of TREM2 and the phagolysosomal pathway in Mn‐treated microglia. CUT&Tag sequencing and qPCR assays demonstrated that 25 µm Mn exposure markedly increased YY1 binding at loci controlling expression of *Trem2*, *Rab7*, and *Lamp1*. To further validate the functional role of YY1, we established a *Yy1*‐KD microglial cell line and ex vivo brain slice model proved that YY1 directly participates in Mn‐induced microglial over‐phagocytosis by transcriptionally activating expression of TREM2 and key molecules associated with the phagolysosomal pathway, thereby driving excessive activation of microglial phagocytic function and lead to abnormal synaptic remodeling. Under 50 µm Mn exposure, the binding affinity of YY1 to its target genes (including *Trem2*, *Rab7*, and *Lamp1*) was markedly reduced compared with this affinity under 25 µm Mn exposure. This reduction did not result from altered YY1 abundance, as YY1 mRNA expression, nuclear accumulation, and protein levels were not significantly different in microglia exposed at the 25 or 50 µm Mn doses. YY1 is a multifunctional zinc finger DNA/RNA‐binding transcription factor, and its regulatory activity is highly sensitive in 170–200 amino acid region posttranslational modifications, particularly acetylation and deacetylation, which modulate its DNA‐binding capability [61, 62, 63]. Based on this evidence, we speculated that the diminished binding of YY1 observed under 50 µm Mn exposure might be attributable to Mn‐induced alterations in YY1 acetylation status.

Previous studies have shown that Mn exposure upregulates HIF1 expression [43], following relationships between HIF1 and YY1 revealed by analyses of JASPAR and GEPIA datasets provided further support for a model in which HIF1α activity promotes YY1 transcription and accumulation under Mn exposure. Moreover, we generated *Hif1α*‐KD microglial cells, demonstrated that HIF1α is responsible for Mn‐induced transcription of *Yy1*. Interestingly, our ChIP assays further revealed that exposure to 25 µm Mn enhanced the binding of HIF1α to the *Yy1* promoter, as well as the binding of YY1 to the *Trem2* promoter. By contrast, no significant change was observed in the binding of HIF1α to the *Trem2* promoter under the same conditions. These findings provide mechanistic evidence that Mn may induce HIF1α expression which promote *Trem2* transcription indirectly through transcriptional activation of *Yy1*, rather than by directly binding to the *Trem2* promoter. YY1 accumulation under Mn exposure might be associated with alterations to ubiquitin‐mediated degradation. Pretreatment of cells with CHX, followed by immunoprecipitation of YY1 revealed that Mn exposure significantly reduced YY1 ubiquitination and suppressed YY1 ubiquitin‐dependent degradation. Using the UbiBrowser 2.0 database, we identified SMURF2 as a putative E3 ubiquitin ligase for YY1, consistent with previous reports implicating SMURF2 in YY1 degradation [63]. Experiments involving overexpression of *Smurf2* in microglia revealed that YY1 undergoes SMURF2‐dependent ubiquitin‐mediated degradation. However, Mn exposure did not affect *Smurf2* mRNA or protein expression, excluding an interaction of Mn with SMURF2 transcription or translation. Molecular docking analyses identified multiple potential Mn^2^^+^ interaction sites within SMURF2, suggesting a potential structural impact. Collectively, these findings demonstrated that Mn‐induced YY1 accumulation in microglia is driven by a dual regulatory mechanism: enhanced transcriptional activation via HIF1α and concomitant dysfunction of SMURF2‐mediated ubiquitin–proteasome degradation.

Despite the comprehensive evidence provided in this study, several limitations should be acknowledged. First, although our data support the involvement of the HIF1α–YY1 axis and SMURF2‐mediated YY1 ubiquitination in Mn‐induced microglial dysfunction, the effects of early‐life Mn exposure on HIF1α and SMURF2 were mainly characterized in vitro and ex vivo. Further studies in vivo and validation in primary microglial cells will are supposed to validate these upstream regulatory mechanisms in the intact brain. The potential effects of Mn ions on SMURF2 conformation and/or functional activity were not investigated in this study. Although this possibility cannot be excluded, future studies are warranted to determine whether Mn directly modulates SMURF2 and contributes to Mn‐induced neurotoxicity. Second, while our ex vivo slice culture experiments supported a critical role for TREM2 in mediating Mn‐induced microglial alterations, the lack of *Trem2* gain‐ or loss‐of‐function mouse models limits direct validation of TREM2‐dependent mechanisms under physiological conditions. Third, our analyses mainly focused on young adulthood after early‐life Mn exposure, whereas longitudinal assessments across earlier developmental stages are still lacking. Future time‐course studies will help define the progression from early microglial alterations to persistent synaptic and behavioral deficits. Finally, although microglial phagocytic activity was reduced at the higher Mn dose (11 mg kg^−1^) compared with the lower dose (1.1 mg kg^−1^), suggesting a non‐linear dose‐dependent effect, the biological basis of this phenomenon may involve multiple cellular adaptations under different exposure levels. Future studies integrating complementary functional assays and in vivo genetic models will help further clarify the mechanisms underlying the dose‐dependent regulation of microglial phagocytic function by Mn.

## 4 Conclusion

Our study demonstrated that early‐life environmental Mn exposure impairs long‐term memory and learning ability and leads to abnormal behavior in young adulthood mice male mice, and we identified excessive activation of microglial phagocytosis and phagolysosomal pathway resulting in aberrant hippocampus synaptic remodeling, driven by the YY1/TREM2 axis, as a key mechanism underlying these deficits. The molecular mechanisms behind these phenomena involve both enhanced transcriptional activation via HIF1α and defective SMURF2‐mediated ubiquitin–proteasome degradation, which contribute to Mn‐induced YY1 accumulation in microglia. Our findings not only provided new insight into how changes to microglia upon environmentally relevant Mn exposure during early neurodevelopment alter neurobehavioral outcomes, but it also revealed potential molecular targets for future strategies aimed at preventing or mitigating microglia‐driven neurodevelopmental disturbances.

## 5 Experimental Section

### 5.1 Animal and Treatments

C57BL/6 mice (8 weeks old) were obtained from the Laboratory Animal Center at China Medical University and raised under identical conditions. Animal procedures were performed in accordance with the Regulations on the Administration of Laboratory Animals and were approved by the Animal Welfare and Ethics Committee of China Medical University (No. CMU20240556). All animal experiments were conducted in compliance with the 3R Principles.

Early‐life Mn‐exposure solutions consisted of 0.9% NaCl and MnCl_2_·4H_2_O (Sigma Aldrich, USA) at doses of 0, 0.11, 1.1, and 11 mg kg^−1^ day^−1^, representing control and environmentally relevant low, medium, and high levels of early‐life Mn exposure. Dose selection was based on multiple considerations. The estimated daily adult human Mn consumption through water, food, and other sources (1.2 mg day^−1^) served as reference for the low dose [26]. Allometric calculations were performed to identify the equivalent value for mice (0.11 mg kg^−1^ day^−1^). The medium Mn‐exposure dose (1.1 mg kg^−1^ day^−1^) was calculated based on environmental Mn contamination reported in a Chinese drinking reservoir (4.11 mg L^−1^) [32], and the high dose (11 mg kg^−1^ day^−1^) was determined in reference to the highest reported environmental Mn concentration (37.202 mg L^−1^), which was found in canal water sediment in Nigeria [33]. Mice were exposed to Mn through the entire early life, from conception through lactation, until weaning on postnatal day 21 (PND 21).

Twenty‐four 8‐week‐old C57BL/6 mice (8 male, 16 female) were randomly assigned to four experimental groups, corresponding to control and low, medium, and high doses. Both male and female mice were exposed to Mn for 60 days via oral gavage once daily prior to mating. After mating, the female mice continued to be exposed to Mn for 42 additional days throughout gestation (21 days) and lactation (21 days). When the offspring reached young adulthood mice (PND 60), neurobehavioral assays were performed on 10 male and 10 female offspring randomly selected from each of the four groups. Then the male offspring were subjected to follow‐up experiments after the behavioral assays.

In some cases, the microglial activation inhibitor minocycline (Mino; MCE, USA) was used in order to investigate whether Mn‐induced microglial activation led to synaptic remodeling. Forty male offspring mice were randomly divided into four experimental groups: control, medium Mn exposure without Mino treatment, Mino treatment alone, and medium Mn exposure with Mino treatment. These mice were subjected to early‐life Mn exposure as described above, and relevant groups were treated with Mino (10 mg kg^−1^) via intraperitoneal injection once every 3 day, beginning on PND 6 and ending on PND 21.

### 5.2 Animal Neurobehavioral Assessments

All behavioral data were collected and analyzed using EthoVision XT tracking software (Noldus, Netherlands). The Morris water maze test was used to assess spatial learning and memory of young adulthood mice following Mn exposure. Latency to escape, time spent in the target quadrant and the number of crossings over the former platform location were quantified as indices of spatial learning and memory retention. The step‐down inhibitory avoidance test was conducted to evaluate long‐term memory. and latency to step down and the number of step‐down events in test sessions were recorded as indicators of long‐term memory. Locomotor activity and anxiety were examined using the open‐field test. Therefore, the total time spent in the central area, average movement speed (cm s^−1^), and the total movement distance were recorded for evaluation of anxiety.

The mice underwent Morris water maze test, a 3‐day habituation phase in a circular pool (diameter, 100 cm) containing a hidden escape platform (diameter, 10 cm) submerged 1 cm below the surface. Animals that failed to reach the platform on the last day of habituation were excluded. During acquisition, each mouse was released into the pool and allowed up to 60 s to locate the platform. Escape latency was recorded as the primary measure of learning. Mice remaining on the platform for 10 s after each trial were included in analyses, and those unable to find the platform within the allotted time were guided to it. The training stage continued for five consecutive days. Twenty‐four hours after the last acquisition session, the probe test was conducted with the platform removed. The step‐down inhibitory avoidance test for assessments of long‐term memory, a cubic chamber (diameter: 5 cm) was established with an insulated rubber platform and a floor with bars connected to a shock generator. Mice were placed on the platform and allowed to explore for 300 s. During training, a mild foot shock was delivered upon stepping down. Retention was assessed 24 h later by returning the mice to the platform. For testing mice locomotor activity and anxiety, open‐field test was performed. A square arena (48 × 48 × 25 cm) was equally separated into 16 smaller squares (12 × 12 cm). Infrared beams along the walls detected movement. Each trial lasted 300 s, beginning when a mouse was first introduced into the arena. Mice would be expected to naturally move to the central area as part of normal exploration; lack of movement to the central area was taken as an indicator of anxiety.

### 5.3 Inductively Coupled Plasma‐Mass Spectrometry (ICP‐MS)

Hippocampal Mn concentrations of offsprings were quantified by ICP‐MS. Tissue samples (50 mg) were weighed and transferred into high‐purity 4% nitric acid (Acros, Denmark) for digestion. After cooling and dilution to 3 mL with deionized water, 100 µL of the solution was further diluted to 2.5 mL for analysis. Mn concentrations were quantified using an Agilent 7700× ICP‐MS (Agilent Technologies, USA) and calculated based on a standard curve.

### 5.4 Golgi–Cox Staining

Synaptic structural remodeling in the offsprings hippocampus was examined using the Hito Golgi–Cox OptimStain PreKit (Hitobiotec Corp, USA) following the manufacturer's protocol. Briefly, brain or organotypic hippocampal slices were immersed in Golgi–Cox solution for 24 h, and the solution was replaced with fresh solution. Slice were then incubated in the dark at room temperature for an additional 14 day, with one final solution change 12 h before processing. Samples were rinsed three times in distilled water, embedded in agarose, and then sectioned at 120 µm thickness. Sections were mounted on gelatin‐coated slides and dried overnight in the dark. The following day, slides were incubated with concentrated mixed staining solution for 10 min, rinsed in distilled water three times, and subjected to graded ethanol dehydration (50%, 70%, 95%, and 100%; 5 min each). Sections were cleared in xylene twice (5 min each), and cover slips were affixed with neutral balsam. Images were acquired using a Nikon 80i microscope, and dendritic and spine analyses in the hippocampal CA1 region were performed with Fiji software (v2.1.0).

### 5.5 Immunofluorescence Assay

The expression of IBA1 (Invitrogen, USA), TMEM119 (CST, USA), TREM2 (Abcam, USA), CD68 (Invitrogen, USA), PSD95 (CST, USA), VGLUT1 (CST, USA), VGAT (CST, USA), and Gephyrin (Invitrogen, USA) in offsprings brain sections, as well as YY1 (CST, USA), LAMP1 (CST, USA), and RAB7 (CST, USA) in cultured cells, was examined by immunofluorescence staining using a confocal microscope. For both tissue sections and cultured cells, samples were fixed with 4% paraformaldehyde (Beyotime, China) for 15 min at room temperature and permeabilized with 0.5% Triton X‐100 (Merck, USA) for 20 min. After blocking with 5% bovine serum albumin (BSA, Gibco, USA) for 1 h, samples were incubated overnight at 4°C with the appropriate primary antibodies, followed by incubation with fluorescently labeled secondary antibodies for 1 h at room temperature. Same experimental indicators were acquired under identical imaging conditions.

For quantification, all analysis in blind manner, and both in vivo or vitro quantification of YY1, CD68, and TREM2 intensities within microglia maker IBA1^+^ regions of interest (ROI) were measured by Fiji (v.2.1.0). For in vitro experiments, nuclear YY1 expression in microglia was quantified using Fiji (v.2.1.0). The DAPI channel was used to generate nuclear ROIs by automated segmentation, and nuclear boundaries were defined accordingly. Mean YY1 fluorescence intensity within the nuclear ROIs was subsequently measured using Fiji (v.2.1.0). The colocalization between LAMP1 and RAB7 was evaluated in Fiji (v.2.1.0) by calculating Pearson's correlation coefficient.

### 5.6 Multiplex Immunofluorescence Assay

To examine the phagocytosis and phagolysosome pathways in microglia, brain sections were processed using a TSA immunofluorescence kit (Immunoway, USA). Brain sections were rinsed three times with phosphate‐buffered saline (PBS) at room temperature, permeabilized with 0.5% Triton X‐100 for 20 min, and treated with endogenous peroxidase blocking buffer (Beyotime, China). After blocking with 5% BSA for 1 h, the sections were incubated overnight at 4°C with the first set of primary antibodies. Following PBS washes, the samples were incubated with the corresponding secondary antibodies and subsequently labeled with iF‐488. After washing and another blocking step with 5% BSA for 1 h, the second set of primary antibodies was applied overnight at 4°C, followed by secondary antibodies and iF‐555 labeling. Finally, the third set of primary antibodies was added overnight, and detection was performed using the respective secondary antibodies and iF‐647. Nuclei were counterstained with DAPI, and images were acquired using a Nikon A1R confocal laser scanning microscope.

For analyses of phagocytosis, Z‐stack images were obtained with a step size of 0.2 µm and reconstructed in Imaris software (v10.1). The Surface function was used to quantify the phagocytic volume of PSD95 and VGAT within microglia. To evaluate the phagolysosomal pathway, ROI were defined based on immunostaining for the microglia marker IBA1, and the fluorescence intensities of LAMP1 and RAB7 were quantified with Fiji software (v.2.1.0).

### 5.7 3D Reconstruction Assays

For 3D reconstruction and quantitative analyses of microglia morphology, phagocytic activity as well as excitatory and inhibitory synaptic quantification, images were acquired by Nikon A1R confocal microscope in Z‐stack (step size: 0.2 µm), visualized and analyzed by Imaris software (v10.1, Bitplane). And three microglia from field of per mice were analyzed. Raw Z‐stack images were acquired under identical imaging settings across all experimental groups and imported into Imaris for 3D reconstruction. All parameters were kept constant across groups, and analyses were conducted in a blinded manner.

For microglial 3D morphological reconstruction, the “Surfaces” and “Filament Tracer” modules were used. Soma segmentation of IBA1^+^ microglia was performed using the Surfaces module, while the process architecture was reconstructed using the Filament Tracer module. Prior to segmentation, background subtraction was applied using local contrast enhancement. An absolute fluorescence intensity threshold was initially determined based on control images and then applied uniformly across all experimental groups without further adjustment. Gaussian smoothing (0.3 µm) was applied to reduce high‐frequency noise prior to surface generation. Surface reconstruction was performed using a fixed surface detail setting (0.5 µm), and identical parameters were maintained for all experimental conditions. Microglial soma and processes were automatically segmented and subsequently verified to ensure consistency across datasets. To minimize analytical bias, all image processing and quantifications were performed in a blinded manner. Nonspecific signals were excluded based on intensity thresholding, and fluorescence signals within approximately 0.5 µm of the cell boundary were removed to reduce potential proximity artifacts. Cell to Body Index (CBI) was used to quantify microglial morphological state and was defined as the ratio of soma volume to total cell volume derived from 3D reconstruction:

$$\mathrm{CBI}=\mathrm{Soma}\;\mathrm{volume}/\left(\mathrm{Soma}\;\mathrm{volume}+\mathrm{Process}\;\mathrm{volume}\right)$$

Microglial phagocytic activity was analyzed using the “Surface” module. IBA1^+^ microglial surfaces were reconstructed and overlaid with engulfed synaptic markers (PSD95, VGAT) to quantify internalized fluorescent volume. The same thresholding parameters and surface smoothness settings were applied across all groups. Phagocytic volume was extracted as the colocalized volume between microglial and synaptic puncta.

Synaptic density was quantified using the “Spots” function. Excitatory (PSD95/VGLUT1) and inhibitory (VGAT/Gephyrin) synaptic puncta were detected using a fixed spot diameter of 0.5 µm and control‐derived intensity threshold applied consistently across all groups. All detection parameters, including background subtraction and quality thresholds, were kept identical across datasets. Synaptic puncta number and fluorescence intensity were quantified within predefined regions of interest.

### 5.8 Transmission Electron Microscopy

Mice hippocampal tissue blocks (1 mm^3^) were fixed in 2.5% glutaraldehyde followed by 1% osmium tetroxide, dehydrated through graded ethanol and acetone, embedded in epoxy resin, and polymerized. Ultrathin sections (60 nm) were double stained with 3% uranyl acetate and lead citrate. Microglial ultrastructure was examined using a Hitachi H‐7650 transmission electron microscope, and lysosomes and phagosomes were quantified.

### 5.9 Single Nucleus RNA Sequencing and Analysis

Single‐cell suspensions were prepared from the brain tissues of three male mice per group at PND 60, and the samples were submitted to Personalbio (Shanghai, China) for single nucleus RNA sequencing (snRNA seq). Nuclei were isolated from frozen hippocampal tissues according to a standard protocol (10× Genomics), and data visualization was performed using Loupe Browser (https://www.10xgenomics.com/cn/products/loupe‐browser). Sequencing reads were processed and aligned with the Cell Ranger Single‐Cell Software Suite (v3.0.2), and downstream analyses were conducted using the Seurat package (v5.0) in R. To ensure data quality, low‐quality cells were filtered out, followed by normalization, scaling, and identification of 2000 highly variable genes via the “FindVariableFeatures” function. Upon clustering and visualization using UMAP and t‐SNE, a total of 1924 microglia were identified from the control group, and a total of 2060 microglia were identified from the Mn‐exposure group. Subpopulations of microglia were defined by reapplying Seurat clustering within each major cell type, with 10 microglia subpopulations ultimately obtained.

DEGs were identified using the “FindMarkers” function in Seurat based on the Wilcoxon rank‐sum test with default parameters. Genes detected in more than 25% of the cells within a cluster and displaying an average log |fold change| greater than 1 were considered as DEGs. Functional enrichment analyses of DEGs were performed using the clusterProfiler R package, incorporating GO and KEGG annotation frameworks. Pathways with adjusted *p* < 0.05 were regarded as significantly enriched. To capture the dynamic trajectories of cellular differentiation, pseudotime analyses were conducted using Monocle3 (v1.3.1). Transcriptional regulatory networks were reconstructed with SCENIC (https://scenic.aertslab.org) in R, thereby revealing transcription factor–target co‐expression modules and associated regulons. To further elucidate molecular interactions, PPI networks were generated using STRING (v12.0) and visualized with Cytoscape (v3.10.4). The cytoHubba plugin employing the MCC ranking algorithm was subsequently used to identify the top 10 hub genes within the network.

### 5.10 Organotypic Hippocampal Slice Culture

Offspring of control‐ and Mn‐treated mice were sacrificed at PND 5 using a procedure that was approved by the Animal Welfare and Ethics Committee, China Medical University (No. CMU20251056). Brains were rapidly removed and placed in ice cold Hank's Balanced Salt Solution (HBSS, Gibco, USA) supplemented with glucose (7.5 mg mL^−1^, Gibco, USA). The organs were embedded in sterile 3% agarose molds, mounted ventral side up on the stage of a vibrating microtome, and sectioned coronally at a thickness of 300 µm to obtain hippocampal slices.

Six‐well plates were prepared by placing porous membrane inserts (Merck, USA) into each well with 1 mL slice culture medium, which consisted of 50% high‐glucose Dulbecco's modified Eagle medium (DMEM) (Gibco, USA), 25% HBSS, 24% heat‐inactivated horse serum (Gibco, USA), and 1% penicillin–streptomycin (Gibco, USA). The membrane inserts were equilibrated in a 37°C, 5% CO_2_ incubator for 1 h prior to slice placement. After positioning the slices on the membrane inserts, excess medium was aspirated so that the medium surface just reached the bottom of the slices without submerging them; the upper surfaces of the slices remained exposed to the incubator atmosphere. Medium was partially replaced (50%) daily and slices were maintained for 20 days, during which morphological maturation and neurite outgrowth from the slice margins were visually monitored daily for the first 7 days, and the slices underwent additional treatments after 10 days of incubation and followed *Trem*‐OE, *Trem2*‐KD, and *Yy1*‐KD brain slice were constructed for continued assessments.

### 5.11 Microglial Targeted Gene Overexpression or Knockdown Establishment in Brain Slices

To manipulate *Trem2* and *Yy1* expression specifically in microglia within organotypic brain slice cultures, microglia‐specific adeno‐associated viral vectors were applied during the culture period to generate *Trem2* knockdown (*Trem2*‐KD), *Trem2* overexpression (*Trem2*‐OE), and *Y* *y1* knockdown (*Yy1*‐KD) slices.

All viral vectors were constructed and packaged by GeneChem Biotech Co., Ltd. (Shanghai, China) using the engineered microglia‐targeting AAV serotype MG.1.2 and the Iba1 promoter to achieve microglia‐specific expression. The complete vector backbone (Iba1‐promoter‐EGFP‐mir155(MCS)‐WPRE‐miR‐9.T‐miR‐129‐2‐3p.T‐SV40p) for *Trem2* knockdown, an AAV vector carrying EGFP under the control of the Iba1 promoter and *Trem2*‐targeting shRNA (target sequence: GACCCTCTAGATGACCAAGAT) was used (vector: GV858; titer: 4.74 × 10^12^ vg mL^−1^). For *Trem2* overexpression, the complete vector backbone (Iba1‐MCS‐3flag‐FT2A‐GFP‐miR‐9.T×4‐miR129‐2‐3p.T.) enabled a microglia‐specific AAV vector encoding the mouse *Trem2* transcript (NM_031254.4) together with GFP reporter was used (vector: GV884; titer: 5.0 × 10^12^ vg mL^−1^). For *Yy1* knockdown, the complete vector backbone (Iba1‐promoter‐EGFP‐mir155(MCS)‐WPRE.miR‐9.T.miR‐129‐2‐3p.T.SV40p), an AAV vector carrying EGFP and *Yy1*‐targeting shRNA (target sequence: CGACGGTTGTAATAAGAAGTT) under the Iba1 promoter was used (vector: GV858; titer: 5.0 × 10^12^ vg mL^−1^).

Organotypic brain slices were cultured as described above and transduced on day 10 in vitro by directly applying the corresponding AAV suspension to the culture medium. Viral particles were maintained in contact with the slices for 72 h before replacing the medium. Viral dosage was selected according to the manufacturer's recommendations (1 × 10^12^ viral genomes per preparation) to ensure efficient transduction in organotypic slices. Transduction efficiency and microglial specificity were verified by immunofluorescence staining, and representative images are provided in Figures S7 and S11. The efficiency of *Trem2* or *Yy1* manipulation was further confirmed by immunofluorescence analysis of target protein expression within IBA1^+^ microglia. And the immunofluorescence analysis was performed using whole‐ slice preparations.

### 5.12 Lactate Dehydrogenase (LDH) Assay

LDH release was quantified to evaluate Mn‐induced cytotoxicity in mice organotypic brain slice cultures. Brain slices were prepared and maintained ex vivo under standard culture conditions, and experiments were initiated on day 13 in vitro. At this stage, slices were exposed to 100 µm Mn for 0, 6, 12, 24, or 48 h. After treatment, the culture medium was collected from each well, and LDH activity was determined using a commercial LDH Cytotoxicity Assay Kit (Beyotime, China) in accordance with the manufacturer's instructions. Absorbance was measured at 490 nm with background correction at 600 nm using a microplate reader. LDH release was normalized to the untreated control and expressed as LDH release (% of control).

### 5.13 Cell Culture

The murine microglia cell line SIM A9 was obtained from ATCC (USA; CRL‐3265). This cell line is derived from mouse primary microglia and retains characteristic microglial morphology and responsiveness. Cells were cultured in DMEM/F‐12 (Gibco, USA) supplemented with 10% BSA, 5% heat‐inactivated goat serum (Gibco, USA), and were maintained at 37°C in a humidified incubator with 5% CO_2_.

To establish stable knockdown or overexpressing cell lines, SIM A9 cells in logarithmic growth phase were transfected with specific lentiviral particles according to the manufacturers’ protocols. Lentiviruses directing the expression of *Yy1* shRNA (sc‐36864‐V, Santa Cruz) and *Hif1a* shRNA (sc‐44308‐V, Santa Cruz) were obtained from Santa Cruz Biotechnology (USA). A recombinant lentiviral vector for overexpression of mouse *Smurf2* (NCBI ID: NM_025481.3) was constructed and packaged by GeneChem Biotech Co., Ltd. (Vector: GV341, Shanghai, China), microglia were transduced with the lentiviral particles for 72 h (viral titer: 1 × 10^8^ TU mL^−1^) and selected with puromycin to generate *Smurf2*‐overexpressing microglial cells, and transduction efficiency was confirmed by RT‐qPCR (Table S1) and Western blot analyses.

For Mn exposure, cells lines were incubated with Mn at final concentrations of 25 or 50 µm for 24 h in DMEM/F‐12 medium containing 5% BSA. After treatment, cells were harvested for subsequent assays.

### 5.14 CCK‐8 Assay

To assess the cytotoxic effects of Mn exposure in vitro, SIM A9 cells were plated in 96‐well plates at a density of 5 × 10^3^ cells per well and allowed to adhere for 24 h prior to treatment. To examine the dose‐ and time‐dependent effects of Mn on microglial viability, cells were exposed to 5, 25, 50, or 100 µm Mn for 3, 6, 12, 24, or 48 h, respectively. Following exposure, 10 µL of CCK‐8 reagent (Dojindo, Japan) was added to each well and incubated for 1 h at 37°C under standard culture conditions. The optical density (OD) at 450 nm was measured using a microplate reader, with culture medium serving as the blank control. The mean OD was used to calculate cell viability according to the following formula:

$$\begin{array}{cc} Cell\;Viability\;\left(\%\right) & =\left(ODtreatment-ODblank\right)/\\ & \;\left(ODcontrol-ODblank\right)\times 100\% \end{array}$$

### 5.15 Flow Cytometry

Microglia were seeded in 6‐well plates and cultured for 24 h before exposure to Mn (25 or 50 µm) for an additional 24 h. After treatment, cells were harvested, resuspended, and washed three times with PBS. The cells were then fixed with 4% paraformaldehyde for 10 min and permeabilized with 0.5% Triton X‐100 for 10 min at room temperature. Subsequently, cells were incubated with CD86‐FITC (Invitrogen, USA) and CD206‐PE (Invitrogen, USA) antibodies in dark room temperature for 30 min. After washing with PBS, the cells were resuspended in 400 µL of PBS and immediately analyzed by BD FACSCanto II flow cytometer. The activation state of microglia was evaluated by calculating the ratio of CD86^+^CD206^−^ to CD86^−^CD206^+^ cells.

### 5.16 Phagocytosis Assay

Microglia phagocytic capacity was assessed in vitro with a Phagocytosis Assay Kit (Cayman Chemical, USA) following an optimized manufacturer's protocol. Briefly, cells were treated with Mn and subsequently incubated with FITC‐labeled beads (1:1000) for 1 h at 37°C. Fluorescence from beads which not phagocytosis by microglia or only bound to the cell surface was quenched by exposure to trypan blue quenching solution for 2 min prior to DAPI staining. Images of phagocytosed beads were acquired with a Nikon A1R confocal microscope. For 3D reconstruction, Z‐stack images were collected at 1 µm intervals and rendered using Imaris (v10.1). Phagocytosed beads volume within microglia per field were quantified using the Surface function of Imaris.

### 5.17 Lysosomal Activity Assays

Lysosomal abundance was visualized following staining of living microglial cells with 50 nm LysoTracker Red (Beyotime, China) for 60 min at 37°C in culture medium. Lysosomal acidity was visualized following staining of cells with 1 µm LysoSensorYellow/Blue (Invitrogen, USA) for 2 h at 37°C. The activity of acidic organelles was visualized following staining of cells with 5 µm acridine orange (MCE, USA) for 1 h at 37°C. After incubation, fluorescence images were acquired using a confocal laser scanning microscope, and the mean fluorescence intensities were quantified by Fiji software (v.2.1.0). In the case of acidic organelle activity, red fluorescence was considered to represent acidic vesicular compartments.

### 5.18 CUT&Tag Assay

CUT&Tag assays were performed using the NovoNGS CUT&Tag 4.0 High‐Sensitivity Kit (Novoprotein, China). The fcCUT&Tag protocol was applied to assess binding of YY1 to cognate DNA. DNA libraries were prepared following the manufacturer's instructions. Briefly, collected cells were fixed with formaldehyde to cross‐link transcription factors to DNA, followed by permeabilization. The samples were incubated with activated ConA beads, resuspended with a YY1 primary antibody (CST, USA), and incubated overnight at 4°C. After washing, anti‐mouse IgG H&L (Novoprotein, China) was applied for 1 h, followed by incubation with ChiTag (pAG‐Transposome) for 1 h at room temperature to perform tagmentation. DNA fragments were extracted using Tagment DNA Extract Beads, amplified by PCR, and purified according to the manufacturer's guidelines. Library quality was verified prior to sequencing and CUT&Tag qPCR was performed to validate the enrichment of target genes. The primers used for qPCR are listed in Table S2.

Sequencing reads were processed using fastp (v0.22.0) for quality control and adapter trimming. Clean reads were aligned to the reference genome using bowtie2 (v2.4.1), and peaks were identified with MACS3 (v3.0.0a7) using the narrow‐peak mode and a threshold of *q* < 0.05. Peak annotation was conducted with ChIPseeker (v1.34.1). Signal profiles were visualized and heatmaps were created with Deeptools (v3.5.1). Merged peak files were generated and quantified using bedtools (v2.30.0) and featureCounts (v2.0.3), respectively. Differential peak analysis was performed with DESeq2 (v1.38.3), defining significance as |log_2_FoldChange| > 1 and *p* < 0.05. Functional enrichment of differential peak‐associated genes was conducted using topGO (v2.50.0) for GO analysis and clusterProfiler (v4.6.0) for KEGG pathway analysis (*p* < 0.05). IGV (v2.19.7) visualized YY1 binding sites on target genes.

### 5.19 RT‐qPCR

Briefly total RNA was isolated from microglia by TransZol kit (TransGen, China). Equal amounts of RNA were reverse transcribed into cDNA using the HiScript III RT SuperMix for qPCR (Vazyme, China). Quantitative real‐time PCR was subsequently performed with ChamQ SYBR qPCR Master Mix (Vazyme, China) on an Applied Biosystems 7500 Real‐Time PCR System (Thermo Fisher Scientific, USA). The primer sequences used for amplification in Table S1. The thermal cycling conditions were as follows: initial denaturation at 95°C for 5 s, followed by 40 cycles of denaturation at 95°C for 10 s and annealing/extension at 60°C for 30 s. Relative mRNA expression levels were calculated using the 2^−ΔΔCT^ method, with *Gapdh* serving as the internal control.

### 5.20 Immunoprecipitation (IP), Co‐Immunoprecipitation (Co‐IP)

For microglial IP and CoIP tests, Pierce Classic Magnetic IP/Co‐IP kit (Thermo Scientific, USA) were applied. In short, cells were lysed in ice cold with YY1 (CST, USA) or SMURF2 (Invitrogen, USA) antibodies for IP overnight at 4°C, antibody complex were banded to Protein A/G magnetic beads for 1 h in room temperature. After IP Lysis/Wash buffer were used for washing, antibody complex eluted, then separated by sodium dodecyl sulfate‐polyacrylamide gel electrophoresis (SDS–PAGE) and analyzed by Western blotting to assess protein–protein interactions.

### 5.21 Western Blotting

Microglia or hippocampal tissues were lysed in RIPA buffer (Beyotime, China) supplemented with protease inhibitors. Obtaining of cell nuclear proteins was performed according to the nuclear protein extraction kit (Beyotime, China) manufacturer's manual. Equal amounts of protein (20 µg) were resolved by SDS–PAGE and transferred onto PVDF membranes (Absin, China). The membranes were blocked with 5% BSA for 1 h at room temperature and then incubated overnight at 4°C with the following primary antibodies: β‐actin (Proteintech, China), Histone H3 (CST, USA), TREM2 (Abcam, USA), LAMP1 (CST, USA), RAB7 (CST, USA), YY1 (CST, USA), HIF1α (CST, USA), SMURF2 (Invitrogen, USA), and anti‐Ubiquitin (Invitrogen, USA). After washing, membranes were incubated with horseradish peroxidase‐conjugated secondary antibodies which including goat anti‐mice IgG (Abmart, China) or goat anti‐rabbit IgG (Abmart, China). Protein bands were detected using an enhanced chemiluminescence substrate and visualized with an Azure C500 imaging system. β‐actin served as the cytosol internal loading control, while Histone H3 served as nuclear internal loading control.

For ubiquitination analysis, cells were pretreated with MG132 10 µm (MCE, USA) for 4 h and cycloheximide (MCE, USA) for 50 µg mL^−1^ for the indicated time periods prior to collection, to block proteasomal degradation or protein synthesis, respectively. The lysates were then subjected to IP with YY1 (CST, USA) antibody as described above. The precipitated complexes were analyzed by Western blotting using anti‐Ubiquitin (Invitrogen, USA) to detect YY1 proteins ubiquitinated forms. This assay was used to evaluate changes in the ubiquitination level of YY1 under Mn exposure.

### 5.22 Chromatin Immunoprecipitation (ChIP) Assay

Cells were fixed with 1% formaldehyde for 10 min to crosslink chromatin‐bound HIF1α or YY1 with DNA. The crosslinking reaction was quenched, and chromatin was sheared into 200–500 bp fragments by sonication. The cell lysates were then incubated overnight at 4°C with anti‐HIF1α (CST, USA) or anti‐YY1(CST, USA), anti‐IgG (Abcam, USA) was used as a negative control, 1% of the input sample was used as a positive control. Subsequently, Dynabeads Protein G (Invitrogen, USA) was added and incubated for 2 h to capture the immune complexes. After reversal of crosslinking and DNA purification, the precipitated DNA fragments were analyzed by ChIP‐qPCR. The primer listed in Table S3.

### 5.23 Enzyme‐Linked Immunosorbent Assay (ELISA)

The levels of TNF‐α, IL‐1β, IL‐6, and IL‐10 in microglia culture supernatants were measured by commercially available ELISA kits (TNF‐α, IL‐1β, IL‐6, and IL‐10, Absin, China). Microglia were treated with Mn (25 or 50 µm) for 24 h, after which the culture media were collected and centrifuged to remove debris. The concentrations of cytokines were then determined according to the manufacturer's instructions, and absorbance was measured by a microplate reader. Results According to the standard curve to determine concentration values, then followed compare with the control group.

### 5.24 Statistical Analysis

Statistical analyses were performed using GraphPad Prism software (v9.5.1 for macOS; GraphPad Software). Data are presented as the mean ± SD. Comparisons among multiple groups were conducted using one‐way analysis of variance (ANOVA) followed by the least significant difference (LSD) post hoc test or two‐way ANOVA followed by Tukey's multiple comparisons test. All experiments were independently repeated at least three times. A *p‐*value < 0.05 was considered to indicate statistical significance.

## Author Contributions

**Keyu Chen**: investigation, methodology, formal analysis, writing – original draft. **Bin He**: methodology, data curation, writing. **Kuan Liu**: methodology, validation, writing – review & editing. **Zhuo Ma**: data curation, writing – review & editing, supervision, funding acquisition. **Bin Xu**: writing – review & editing, conceptualization, supervision, funding acquisition. The corresponding author is responsible for ensuring that the descriptions are accurate and agreed by all authors.

## Ethics Statement

This is a preclinical (nonclinical) research study that does not require ethics approval and consent to participate for human subjects. All preclinical animal studies were undertaken on fully approved Institutional Animal Care and Use Committee protocols (see Experimental Section).

## Conflicts of Interest

The authors declare no conflicts of interest.

## Supporting information




**Supporting File**: advs78036‐sup‐0001‐SuppMat.docx.

## Data Availability

Data generated or analyzed during this study are included in the article or its supplemental information files. This paper does not report original code. Any additional information required to reanalyze the data reported in this paper is available from the authors upon request.
