# Supplementary material for: Early‐Life Manganese Overexposure Activates Microglial Phagocytosis through the YY1/TREM2 Axis Leading to Hippocampal Synaptic Remodeling in Mice
Source: Adv Sci (Weinh). 2026 Oct 4:e78036. Online ahead of print. doi: 10.1002/advs.78036 (PMC13634581; doi:10.1002/advs.78036)
Supplement: Supplementary file 1 [file ADVS-9999-e78036-s001.docx]

Supporting Information for

**Early-life manganese overexposure activates microglial phagocytosis through the YY1/TREM2 axis leading to hippocampal synaptic remodeling in mice**

***Authors:*** *Keyu Chen, Bin He, Kuan Liu, Zhuo Ma * & Bin Xu **

*Bin Xu, Zhuo Ma.*

Key laboratory of Environmental Stress and Chronic Disease Control and Prevention (China Medical University), Ministry of Education, Shenyang, Liaoning 110122, China

Program of Environmental Physical Factors and Health, School of Public Health, China Medical University, Shenyang, Liaoning 110122, China

E-mail: bxu10@cmu.edu.cn (B. Xu), zma@cmu.edu.cn (Z. Ma)

*Keyu Chen, Bin He.*

Program of Environmental Physical Factors and Health, School of Public Health, China Medical University, Shenyang, Liaoning 110122, China

*Kuan Liu.*

Department of General Surgery, Shengjing hospital of China Medical University, Shenyang, Liaoning 110004, China

** Co-Corresponding Authors: Zhuo Ma and Bin Xu*

**·Supplemental Figures:**

**Figure S1.** **Early-life manganese exposure has slightly significant impact on the behavior of female offspring, related to Figure 1.** (**A- D**) Morris water maze performance of female or male offsprings under different doses of early-life Mn exposure, and statistical results of female offspring latency to escape (s) and platform location crossed times, female or male offspring time spent in target quadrant (%). (**E, F)** Statistical results of the step-down passive avoidance test in female offspring after early-life Mn exposure. (**G- J**) Open field test performance of female or male offsprings exposed to different doses of Mn during early-life, and related statistical analysis of female offspring time spent in central area (s), female and male offsprings average travel speed (cm/s) and total travel distance (cm). Data were expressed as mean ± SD. For behavioral experiments, *n* = 10 mice per group. One-way ANOVA followed by Fisher’s LSD post hoc test. ^*^*p* < 0.05 ，^**^*p* < 0.01vs Control group.

**Figure S2.** **Hippocampal synaptic remodeling occurs in following male offspring early-life manganese exposure, related to Figure 1.** (**A**) Representative Golgi-cox staining image of offsprings hippocampus and neuron skeleton. Scale bar, 100μm, magnification scale bar, 40μm. (**B)** Sholl analysis of neuronal cytoskeletal morphological alterations. (**C, D)** Immunofluorescence of excitatory synapses and inhibitory synapses, with VGLUT1 (green) and PSD95 (red) as excitatory synapses marker and VGAT (red) and Gephyrin (green) as inhibitory synapses marker, followed the merged fluorescence intensity was measured. Scale bar,10μm. (**E, F)** ES and IS scanned by confocal microscope and reconstructed，the relative co-localization counts of synapses was calculated. Scale bar, 10μm, magnification scale bar, 5μm. Data were expressed as mean ± SD. For in vivo experiments, *n* = 4 mice per group. One-way ANOVA followed by Fisher’s LSD post hoc test. ^*^*p* < 0.05, ^**^*p* < 0.01vs Control group.

**Figure S3.** **Early-life manganese exposure activates microglial phagocytosis function in offspring hippocampal CA1 region, related to Figure 1.** (**A**) Distribution of microglia (IBA1, green) in the hippocampal region. Scale bar, 100μm. (**B**) Microglia in the hippocampus marked by IBA1 (green) and TMEME119 (red), with Pearson colocalization coefficient. Scale bar, 50μm. (**C- E**) 3D reconstruction of microglia and statistical analysis of microglial morphological analysis. Scale bar, 30μm. (**F**) Immunofluorescence images showing IBA^+^ microglia and the activation marker CD68 (red) in the hippocampal region. Scale bar, 50μm, magnification scale bar, 30μm. (**G**) 3D reconstruction was used to evaluate the IBA^+^ microglia phagocytic capacity of PSD95 (red) and VGAT (pink). Scale bar, 10μm. (**H**) Transmission electron microscopy showing changes in phagosomes (yellow) and lysosomes (pink) in microglia within the hippocampal CA1 region. Scale bar, 2μm, magnification scale bar, 500nm. Data were expressed as mean ± SD. For in vivo experiments, *n* = 4 mice per group. One-way ANOVA followed by Fisher’s LSD post hoc test. ^**^*p* < 0.01vs Control group.

**Figure S4. Minocycline inhibits microglial phagocytic activation and alleviated synaptic remodeling, related to Figure 2.** (**A**) Sholl analysis showing microglial morphological complexity under different treatment conditions. (**B**) Quantitation of microglial morphology showing total branch length (μm) under different treatment. (**C**) Representative immunofluorescence images showing microglial activation marker CD68 (red) and quantitative analysis of CD68 fluorescence intensity within IBA1^+^ microglia. Scale bar, 50μm, magnification scale bar, 30μm. (**D, E**) Quantification of colocalized fluorescence intensity in merged excitatory and inhibitory synaptic signals, Scale bar, 10μm. (**F, G**) Colocalization of relative excitatory and inhibitory synapses quantified using 3D reconstruction. Data were expressed as mean ± SD. For in vivo experiments, *n* = 4 mice per group. Data were analyzed using two-way ANOVA followed by Tukey’s multiple comparisons test. ^**^*p* < 0.01 vs Control group. ^#^*p* < 0.05, ^##^*p* < 0.05 vs 1.1mg/kg Mn group.

**Figure S5. TREM2 is central molecule driving phagocytic activity in microglia, related to Figure 3.** (**A**) Relative proportions of microglial subpopulations in Control and Mn (1.1mg/kg) groups. (**B**) Pseudotemporal trajectories of microglia highlighting divergent fates (Fate1 and Fate2) under Mn exposure. (**C, D**) KEGG enrichment of MG1 and MG2 microglia subclusters reveals phagocytic characteristics. (**E**) Protein-protein interaction network of overlapping DEGs between MG1 and MG2 microglia subclusters generated by STRING. (**F**) Overlap of DEGs between published activated microglia and activated microglia from offspring mice. (**G**) Protein–protein interaction network of DEGs from activated microglia in offspring mice constructed using the STRING. (**H**) Identification of the top 10 hub genes and their MCC scores from the interaction network by Cytoscape.

**Figure S6. Early-life Mn exposure induces microglia TREM2 and phagolysosomal pathway activation, related to Figure 3.** (**A, B**) Visualization and quantification of TREM2 (red) expression in hippocampal CA1 IBA1^+^ microglia of offspring mice via immunofluorescence. Scale bar, 40μm, magnification scale bar, 20μm. (**C, D**) Protein expression of TREM2 in the hippocampus of Control and different Mn exposed offspring mice by Western blot, normalized to β-actin and relative to control. (**E- G**) Representative TSA immunofluorescence images showing phagocytic (RAB7, green) lysosomal (LAMP1, red) pathway activation markers in IBA1^+^ microglia (gray) across different treatment groups, along with quantitative analysis of mean fluorescence intensity and area relative to control (%). Scale bar, 50μm. Data were expressed as mean ± SD. For in vivo Western blot analysis, *n* = 3 mice per group; for all other in vivo experiments, *n* = 4 mice per group. One-way ANOVA followed by Fisher’s LSD post hoc test. ^**^*p* < 0.01 vs Control group. ^#^*p* < 0.05, ^##^*p* < 0.05 vs 1.1mg/kg Mn group.

**Figure S7. Ex vivo brain slices culture reveals TREM2 role in Mn-induced abnormal synaptic remodeling, related to Figure 3.** (**A**) Representative brightfield images of offspring brain slices after 10 days of ex vivo culture. Scale bar, 50μm. (**B**) AAV vectors expressing GFP transduced microglia in ex vivo brain slices. Scale bar, 50μm. (**C**) Quantification of microglial transfection efficiency in brain slices, calculated as the percentage of GFP⁺IBA1⁺ cells among total IBA1⁺ cells. (**D**) mRNA expression level of *Trem2* in different treatment of brain slices, normalized to *Gaphd* and relative to control. (**E, F**) Representative immunofluorescence images of TREM2 (green) in microglia (IBA1, red) within ex vivo brain slices from Control and Vector, *Trem2*-KD and *Trem2*-OE groups, along with quantitative analysis of colocalized fluorescence intensity. Scale bar, 50μm, magnification scale bar, 20μm. **(G, H)** Western blot and quantification analysis of TREM2 protein expression under different treatment, normalized to β-actin and relative to control. (**I**) Relative LDH release from brain slices at different time points following 100 µM Mn exposure. (**J - M**) Golgi-Cox staining of brain slices from different treatment groups and quantification of associated dendritic spine analysis. Scale bar, 5μm. (**N**) Representative images of fluorescence and 3D reconstruction of presynaptic VGAT (green) and postsynaptic PSD95 (red) in microglia of *Trem2*-KD and *Trem2*-OE brain slices. (**O**) Z-stack and 3D reconstruction images of excitatory synapses immunolabeled with PSD95 (red)/VGLUT1 (green) and inhibitory synapses immunolabeled with VGAT (red)/Gephyrin (green) in *Trem2*-KD and *Trem2*-OE brain slices, along with Pearson’s colocalization coefficients of merged synaptic signals. Data were expressed as mean ± SD. For Western blot analysis, *n* = 3 biologically independent brain slice preparations; for all other in vivo experiments, *n* = 4 biologically independent brain slice preparations. Data were analyzed using two-way ANOVA followed by Tukey’s multiple comparisons test. ^**^*p* < 0.01vs Control group.

**Figure S8. Mn treatment contributes to microglia activation, related to Figure 4.** (**A**) Fluorescence images of SIM A9 microglia (IBA1, red) cell line. Scale bar, 50μm. (**B**) Microglia viability under different Mn doses and treatment durations. (**C, D**) Flow cytometry analysis of microglia activation under different concentrations of Mn and corresponding activation level (CD86^+^ CD206^-^/ CD86^-^ CD206^+^). (**E- H**) Quantification of pro-inflammatory (*Il1b, Il 6, Tnf*) and anti-inflammatory (*Il10*) cytokine mRNA levels in microglia exposed to varying doses Mn by RT-qPCR, normalized to *Gaphd* and relative to control. (**I- L**) Pro-inflammatory (IL1β, IL6, TNFα) and anti-inflammatory (IL10) cytokine in different Mn exposure of microglia by ELISA*,* relative to control. (**M**) Phagocytic activity of microglia toward latex beads following treatment with different concentrations of Mn. Scale bar, 20μm. (**N**, **O**) Representative fluorescence images and quantitative analysis of acidic organelle levels in microglia assessed by Acridine Orange staining, relative to control. Scale bar, 20μm. (**P**) Representative fluorescence of phagocytic (RAB7, green) lysosomal (LAMP1, red) pathway activation level of microglia Mn (50μM), with Pearson’s colocalization coefficients. Scale bar, 20μm, magnification scale bar, 10μm. Data were expressed as mean ± SD. For in vitro experiments *n* = 4 independent experiments for all assays. One-way ANOVA followed by Fisher’s LSD post hoc test. ^*^*p* < 0.05, ^**^*p* < 0.01vs Control group, ^#^*p* < 0.05, ^##^*p* < 0.01 Mn (50μM) vs Mn (25μM) group.

**Figure S9. Mn-induced Yy1 upregulation enhances phagocytosis and lysosomal activation, related to Figure 4.** (**A- C**) snRNA sequencing SCENIC transcription factor analysis reveals that Mn exposure induces upregulation of transcription factor YY1. (**D, E**) KEGG and GO enrichment analysis of up-regulated DEGs associated with YY1 binding after Mn exposure in snRNA sequencing data. (**F**) JASPAR database prediction indicates that YY1 potentially regulates *Trem2* expression. (**G**) Co-expression analysis of *Trem2* and *Yy1* in the hippocampus by GEPIA database. (**H**) Volcano plot of DEGs identified by CUT&Tag sequencing after Mn treatment. (**I**) YY1 binding strength at *Rab7* (phagosome marker) and *Lamp1* (lysosome marker) sites following Mn treatment. (**J**) mRNA expression level of *Yy1*, *Trem2*, *Lamp1* and *Rab7* were measured by RT-qPCR, normalized to *Gaphd* and relative to control, *n* = 4 independent experiments. Data were expressed as mean ± SD. One-way ANOVA followed by Fisher’s LSD post hoc test. ^*^*p* < 0.05, ^**^*p* < 0.01vs Control group. ^##^*p* < 0.01 Mn (50μM) vs Mn (25μM) group.

**Figure S10. YY1 regulates lysosomal pathway activation in microglia, related to Figure 5.** (**A**) Brightfield images showing the effects of puromycin selection on microglia and the generation of stable *Yy1*-KD cell lines. Scale bar, 100μm. (**B**) Relative *Yy1* mRNA expression determined by RT-qPCR, normalized to *Gaphd* and relative to control. (**C, D**) Western blot and quantification showing relative YY1 protein levels, normalized to β-actin and relative to control. (**E**) ChIP assay demonstrating YY1 occupancy at the *Trem2* promoter. (**F- H**) LysoTracker Red and PH-LysoSensor Yellow/Blue staining were used to assess lysosomal abundance and function in microglia along different groups, Scale bar, 20μm. (**I, J**) Representative AO staining images of microglia from different treatment groups and corresponding quantitative analysis of fluorescence intensity relative to control. Scale bar, 20μm. (**K**) Representative immunofluorescence images showing colocalization of phagocytic (RAB7, green) and lysosomal (LAMP1, red) markers in microglia in different groups, with colocalization coefficients. Scale bar, 20μm, magnification scale bar, 10μm.Data were expressed as mean ± SD. For in vitro experiments, *n* = 3 independent experiments for Western blot analysis, and *n* = 4 independent experiments for all other assays. Data were analyzed using two-way ANOVA followed by Tukey’s multiple comparisons test. ^**^*p* < 0.01vs Control group. ^##^*p* < 0.01 vs Mn (25μM) group.

**Figure S11. *Yy1* knockdown alleviates Mn-induced TREM2 upregulation and abnormal synaptic remodeling, related to Figure 5.** (**A**) Representative bright-field images of ex vivo brain slices after 10 days in culture. Scale bar, 100 μm. (**B**) AAV vectors expressing GFP transduced microglia in ex vivo brain slices. Scale bar, 50 μm. (**C**) Quantification of microglial transfection efficiency in brain slices, calculated as the percentage of GFP⁺IBA1⁺ cells among total IBA1⁺ cells. (**D, E**) Representative immunofluorescence images validating *Yy1* knockdown efficiency in microglia within brain slices. IBA1 (green) and YY1 (red), together with Pearson’s correlation coefficient analysis and quantification of mean fluorescence intensity of YY1 in IBA1^+^ microglia. (**F, G**) Representative immunofluorescence images showing TREM2 (red) expression in IBA1^+^ microglia following *Yy1* knockdown in brain slices, together with quantification of fluorescence intensity. (**H, I**) Quantification of excitatory and inhibitory synaptic fluorescence intensity in *Yy1*-knockdown brain slices. Data were expressed as mean ± SD. Data are from *n* = 4 biologically independent brain slice preparations. Data were analyzed using two-way ANOVA followed by Tukey’s multiple comparisons test. ^**^*p* < 0.01vs Control group. ^##^*p* < 0.01 vs Mn group.

**Figure S12. Blockade of HIF1α and dysfunction of SMURF2-mediated ubiquitination contribute to Mn-induced accumulation of YY1 in microglia, related to Figure 6.** (**A**) Brightfield images of established *Hif1a*-KD and *Smurf2*-OE SIM A9 microglia cell lines after puromycin selection. Scale bar, 100μm.(**B**) RT-qPCR analysis of *Hif1a* mRNA expression in *Hif1a*-KD microglia, normalized to *Gaphd* and relative to control. (**C, D**) Western blot analysis showing relative HIF1α protein levels in *Hif1a*-KD microglia, normalized to β-actin and relative to control. (**E**) ChIP assay demonstrating HIF1α occupancy at the *Trem2* promoter. (**F**) RT-qPCR analysis of *Smurf2* mRNA expression in *Smurf2*-OE microglia. (**G, H**) Western blot revealing relative SMURF2 protein expression in *Smurf2*-OE microglia, normalized to β-actin and relative to control. (**I**) Co-immunoprecipitation assays revealing the interaction between YY1 and SMURF2. (**J**) Immunoprecipitation analysis demonstrating ubiquitination levels of YY1 under various groups. (**K, L**) Analysis of YY1 protein levels in control and *Smurf2*-OE groups following MG132 treatment by Western blot, normalized to β-actin and relative to control. (**M, N**) YY1 degradation dynamics in Control and *Smurf2*-OE groups at different time points after CHX treatment, normalized to β-actin and relative to control. For in vitro experiments, *n* = 3 independent experiments for Western blot analysis, and *n* = 4 independent experiments for all other assays. Data were analyzed using two-way ANOVA followed by Tukey’s multiple comparisons test. ^*^*p* < 0.05 ^**^*p* < 0.01vs Control group. ^##^*p* < 0.01 vs Control+MG132 group.

**·Supplemental Tables:**

**Table S1：Primer Sequences for RT-qPCR**

| *Gene* | Sequence |
| --- | --- |
| ***Hif1α*** | Forward primer 5’- TCCTTCGGACACATAAGCTCC -3’ |
|  | Reverse primer 5’- GACAGAAAGATCATGTCACCGT -3’ |
| ***Yy1*** | Forward primer 5’- ATGAGAAAGCATCTGCACACC -3’ |
|  | Reverse primer 5’- AGCCTTCGAATGTGCACTGAAA -3’ |
| ***Trem2*** | Forward primer 5’- CTGGAACCGTCACCATCACTC -3’ |
|  | Reverse primer 5’- CGAAACTCGATGACTCCTCGG -3’ |
| ***Smurf2*** | Forward primer 5’- GACATAGGTTCCTGCTGGACAC -3’ |
|  | Reverse primer 5’- CGCCCAtCAAGGGCCCCAAG-3’ |
| ***Lamp1*** | Forward primer 5’- CCAGAGCGTTCAACATCAGC -3’ |
|  | Reverse primer 5’- ACAGGCTAGAGCTGGCATTC -3’ |
| ***Rab7*** | Forward primer 5’- TTTACCCCTTGTCCCCTCTT -3’ |
|  | Reverse primer 5’- ATGGTTGTCCCCATCTTTGA -3’ |
| ***Tnf*** | Forward primer 5’- AGAAGTTCCCAAATGGCCTC -3’ |
|  | Reverse primer 5’- CCACTTGGTGGTTTGCTACG -3’ |
| ***Il1b*** | Forward primer 5’- GCCCATCCTCTGTGACTCAT -3’ |
|  | Reverse primer 5’- AGCTCATATGGGTCCGACAG -3’ |
| ***Il6*** | Forward primer 5’- AGTTGCCTTCTTGGGACTGA -3’ |
|  | Reverse primer 5’- TCCACGATTTCCCAGAGAAC -3’ |
| ***Il10*** | Forward primer 5’- GGTTGCCAAGCCTTATCGGA -3’ |
|  | Reverse primer 5’- TTCAGCTTCTCACCCAGGGA -3’ |
| ***Gapdh*** | Forward primer 5’- GGAGCGAGATCCCTCCAAAAT -3‘ |
|  | Reverse primer 5’- GGCTGTTGTCATACTTCTCATGG -3‘ |

**Table S2：Primer Sequences for CUT＆Tag qPCR**

| *Gene* | Sequence |
| --- | --- |
| ***Rab7*** | Forward primer 5’- AGAGAGAAGGCTCTCTGACC-3‘ |
|  | Reverse primer 5’- CCAGGGTCATCTGAGTGTGA-3‘ |
| ***Lamp1*** | Forward primer 5’- TCTCAGTGAACTACGACACCA -3’ |
|  | Reverse primer 5’- AGTGTATGTCCTCTTCCAAAAGC -3’ |
| ***Trem2*** | Forward primer 5’- CCATCTAGGCCTTAACAT -3’ |
|  | Reverse primer 5’- AAATCACTGGACAGGAAC -3’ |

**Table S3：Primer Sequences for ChIP assay**

| *Gene* | Sequence |
| --- | --- |
| ***Yy1***  ***Trem2*** | Forward primer 5’- TTTTGTGGCTGTTGCACCG -3’ |
|  | Reverse primer 5’- AATCGATCTGTCCGCTGGC -3’  Forward primer 5’- CCATCTAGGCCTTAACAT -3’  Reverse primer 5’- AAAT CACTGGACAGGAAC -3’ |
